# Novel AHR ligand AGT-5 ameliorates type 1 diabetes in mice through regulatory cell activation in the early phase of the disease

**DOI:** 10.3389/fimmu.2024.1454156

**Published:** 2024-09-06

**Authors:** Natalija Jonić, Ivan Koprivica, Stavroula G. Kyrkou, Vasileios-Panagiotis Bistas, Christos Chatzigiannis, Nataša Radulović, Ivan Pilipović, Andjelina Jovanović, Milan B. Jovanović, Mirjana Dimitrijević, Andreas G. Tzakos, Ivana Stojanović

**Affiliations:** ^1^ Department of Immunology, Institute for Biological Research “Siniša Stanković” - National Institute of the Republic of Serbia, University of Belgrade, Belgrade, Serbia; ^2^ Section of Organic Chemistry & Biochemistry, Department of Chemistry, University of Ioannina, Ioannina, Greece; ^3^ Department of Otorhinolaryngology with Maxillofacial Surgery, Clinical Hospital Center “Zemun”, Belgrade, Serbia; ^4^ Faculty of Medicine, University of Belgrade, Belgrade, Serbia; ^5^ Institute of Materials Science and Computing, University Research Center of Ioannina (URCI), Ioannina, Greece

**Keywords:** type 1 diabetes (T1D), aryl hydrocarbon receptor (AhR), T regulatory cell (TREG), insulitis, gut-associated lymphoid tissue (GALT), lamina propria

## Abstract

Type 1 diabetes (T1D) is an autoimmune disease with a strong chronic inflammatory component. One possible strategy for the treatment of T1D is to stimulate the regulatory arm of the immune response, i.e. to promote the function of tolerogenic dendritic cells (tolDC) and regulatory T cells (Treg). Since both cell types have been shown to be responsive to the aryl hydrocarbon receptor (AHR) activation, we used a recently characterized member of a new class of fluorescent AHR ligands, AGT-5, to modulate streptozotocin-induced T1D in C57BL/6 mice. Prophylactic oral administration of AGT-5 reduced hyperglycemia and insulitis in these mice. Phenotypic and functional analysis of cells in the pancreatic infiltrates of AGT-5-treated mice (at the early phase of T1D) revealed a predominantly anti-inflammatory environment, as evidenced by the upregulation of tolDC and Treg frequency, while CD8^+^ cell, Th1 and Th17 cells were significantly reduced. Similarly, AGT-5 enhanced the proportion of Treg and tolDC in small intestine lamina propria and suppressed the activation status of antigen-presenting cells through down-regulation of co-stimulatory molecules CD40, CD80 and CD86. The expression levels of Cyp1a1, controlled by the AHR, were increased in CD4^+^, CD8^+^ and Treg, confirming the AHR-mediated effect of AGT-5 in these cells. Finally, AGT-5 stimulated the function of regulatory cells in the pancreatic islets and lamina propria by upregulating indoleamine 2,3-dioxigenase 1 (IDO1) in tolDC. These findings were supported by the abrogation of AGT-5-mediated *in vitro* effects on DC in the presence of IDO1 inhibitor. AGT-5 also increased the expression of CD39 or CD73 ATP-degrading ectoenzymes by Treg. The increase in Treg is further supported by the upregulated frequency of IL-2-producing type 3 innate lymphoid cells (ILC3) in the lamina propria. Anti-inflammatory effects of AGT-5 were also validated on human tonsil cells, where *in vitro* exposure to AGT-5 increased the proportion of immunosuppressive dendritic cells and ILC3. These results suggest that AGT-5, by stimulating AHR, may promote a general immunosuppressive environment in the pancreas and small intestine lamina propria at the early phase of disease, and thereby inhibit the severity of T1D in mice.

## 1 Introduction

Type 1 diabetes mellitus (T1D) is a chronic autoimmune disease with a rising global incidence. This observation highlights a growing influence of environmental factors on T1D onset and development. T1D is characterized by the destruction of pancreatic β-cells, insulin deficiency and hyperglycemia. T1D, and especially its early onset in children, often leads to serious complications including heart disease, renal disease, circulatory problems, blindness, etc. At present, despite the extensive research of various treatment modalities in animal T1D models, the therapy of T1D is limited to multiple daily insulin injections that, often inadequately, prevent severe hyperglycemia and other long-term complications (1).

A breakdown of self-tolerance to pancreatic β-cell antigens is a hallmark of T1D (2). In T1D, β-cells are destroyed by the autoantigen-specific CD4^+^ and CD8^+^ T effector cells (Teff) leading to insulin deficiency (3). Regulatory T cells (Treg) maintain immune tolerance and exert immunosuppressive activities toward Teff. The main feature of T1D is the imbalance between the Teff and the FoxP3^+^ CD4^+^ Treg (4). Numerous studies have demonstrated that the absence of Treg in mice leads to the development of T1D and that increased activity and number of these cells results in the resolution of T1D (5, 6). Moreover, a reduction in the number or defective function of Treg has been demonstrated in patients with T1D (7, 8).

Key environmental factors associated with T1D susceptibility are the diet and the gut microbiome. A potential mechanistic link between diet, gut microbiome and T1D might be related to the aryl hydrocarbon receptor (AHR), a transcription factor that is activated by many environmental signals (9). The most common biomarker of AHR activity is the expression of Cyp1a1, which belongs to the Cytochrome P450 family of enzymes and is involved in phase I metabolism (detoxification) of numerous endogenous and exogenous compounds. Importantly, AHR activation was found to initiate the transcription of both pro-inflammatory and anti-inflammatory genes in immune cells, depending on the cell type and extracellular environment (9). The anti-inflammatory outcomes of AHR signaling can include, for example, differentiation of type 1 regulatory T cells and FoxP3^+^ Treg (10), stimulation of IL-10 production by inflammatory macrophages (11), and downregulation of IFN-γ and IL-12 secretion by splenic cells (12).

AHR signaling sustains intestinal barrier integrity through the direct effect on epithelial cells and on the immune cells residing in the gut lamina propria or within the epithelium (13). Dietary AHR ligands modulate the abundance of intestinal bacteria whose metabolites are associated with T1D development (14). Supplementation with AHR activators is very effective in rodent models of inflammation including autoimmune diseases such as T1D. Activation of AHR by 2,3,7,8-tetrachlorodibenzo-p-dioxin (TCDD) prevents T1D in NOD mice by increasing Treg population in the pancreatic lymph nodes (15). Also, nanoparticles containing 2-(1’ H-indole-3’-carbonyl)-thiazole-4-carboxylic acid methyl ester (ITE), through activation of AHR in tolerogenic dendritic cells (tolDC), prevent T1D in NOD mice (16). Activation of the AHR by certain ligands was shown to prevent insulitis and Teff development independently of FoxP3^+^ Treg in NOD mice (17).

Having in mind that tolDC, type 3 innate lymphoid cells (ILC3) and Treg exhibit high expression of AHR (9), our aim was to explore the effects of AGT-5, a member of the new class of fluorescent AHR ligands (FluoAHRL) on multiple low dose streptozotocin (STZ)-induced T1D development in mice (18, 19). This compound exerts AHR agonistic effects in both mouse and human cells (20). In addition to emitting in the near-infrared (NIR), enabling bioimaging of AHR, AGT-5 has been demonstrated to stimulate the differentiation, proliferation, and function of Treg *in vitro* (20). This pivotal characteristic positions AGT-5 as a promising candidate for modulating the autoimmune process in T1D.

## 2 Material and methods

### 2.1 AGT-5 synthesis

AGT-5 was synthesized according to the protocol published in Jonić et al. (20). Briefly, synthesis was conducted through a Knoevenagel condensation. Specifically, a solution of 2-(2-methyl-4H-chromen-4-ylidene)malononitrile (1eq) and indole-3-carboxaldehyde (1eq) in acetonitrile, with catalytic amount of piperidine, was stirred under reflux for 16 hours. The crude reaction mixture was purified using silica gel column chromatography with CH_2_Cl_2_ as the eluent, resulting in a pure red product (yield 64%).^1^H NMR (250 MHz, DMSO, δ ppm): 11.96 (d, J = 2.9 Hz, 1H), 8.76 (dd, J = 8.4, 1.4 Hz, 1H), 8.29 – 8.24 (m, 1H), 8.10 – 8.01 (m, 2H), 7.92 (ddd, J = 8.5, 7.1, 1.5 Hz, 1H), 7.80 (dd, J = 8.4, 1.3 Hz, 1H), 7.61 (ddd, J = 8.4, 7.1, 1.3 Hz, 1H), 7.52 (dt, J = 8.1, 0.9 Hz, 1H), 7.33 – 7.21 (m, 3H), 7.13 (s, 1H) (**Supplementary Figure S1**). ^13^C NMR (63 MHz, DMSO, δ ppm): 160.77, 153.24, 152.57, 138.10, 135.41, 134.98, 133.51, 126.31, 125.19, 125.08, 123.43, 121.65, 121.20, 119.31, 118.48, 117.77, 117.04, 114.03, 113.08, 112.98, 104.99, 57.47 (**Supplementary Figure S2**).

The product’s purity underwent further assessment through Agilent analytical HPLC chromatography employing an InfinityLab Poroshell 120 EC-C18 column, sized at 4.6×150 mm, alongside a diode array detector (DAD). Specific resolution was monitored at 254 nm. A gradient solvent system (A: 5%, B: 95% to A: 100%, B: 0%) composed of A: acetonitrile + 0.1% formic acid and B: H2O + 0.1% formic acid was utilized, maintaining a constant flow rate of 1.0 ml/min over a duration of 10 min. The peak for AGT-5 reached its apex at 8.3 min, revealing a purity level of 96% (**Supplementary Figure S3**).

A Xevo G2 Q-TOF mass spectrometer, employed for a direct infusion acquisition utilizing full scan MS with a mass scan range of 50–1200 m/z, operated in positive ESI (ElectroSpray Ionisation) mode. Maximum ion intensity was typically achieved with the following source conditions: a capillary voltage of 3.0 kV, sample cone voltage of 40 V, source temperature of 120**°**C, desolvation temperature of 550**°**C, cone gas flow rate of 100 L h^−^¹, and desolvation gas (N_2_) flow rate of 600 L h^−^¹. MS: m/z for C_22_H_13_N_3_O: calculated 335.11, found 336.11300 [M+H]^+^ (**Supplementary Figure S4**).

### 2.2 Mice and human tissue

C57BL/6 mice were bred and maintained at the Animal Facility at the Institute for Biological Research “Siniša Stanković”- National Institute of the Republic of Serbia, University of Belgrade, with free access to food and water, and hiding structures added for environmental enrichment. All experiments were approved by the Veterinary Directorate of the Ministry of Agriculture, Forestry and Water Management of the Republic of Serbia (App. No 119-01-4/11/2020-09) and were in accordance with the Directive 2010/63/EU on the protection of animals used for scientific purposes. Tonsils were obtained following patient informed consent signing and all procedures were conducted in accordance with the Declaration of Helsinki and approved by the Ethics Committee of Clinical Hospital Center “Zemun”, Belgrade, Serbia (App. No 14/1, date 27/09/2022).

### 2.3 Induction of T1D in C57BL/6 mice and AGT-5 treatment

Male 2-month-old C57BL/6 mice were subjected to T1D induction using multiple low doses of STZ, which were applied intraperitoneally for 5 consecutive days. STZ (38 mg/kg body weight; Sigma-Aldrich, St. Louis, MO, USA) was dissolved in cold 0.1 M citrate buffer (pH 6) right before administration. The powder form of AGT-5 was initially dissolved in DMSO (Sigma-Aldrich), and then in sesame oil. AGT-5 was applied orally (with a metal feeding tube inserted into the proximal esophagus) from the first day of T1D induction and treatment continued for 20 days (prophylactic regime). Molecular weight of AGT-5 is 335.37 g/mol. Each day, AGT-5 was freshly dissolved in DMSO to the concentration of 250 mg/ml (2.5 mg in 10 µl DMSO) and then diluted 1:100 with the sesame oil to obtain the final concentration of 2.5 mg/ml. Mice received approximately 100 μl of the final dilution (10 mg/kg body weight). The control group animals were given the same volume of sesame oil containing only DMSO (1% v/v). Number of mice per group for clinical assessment was 8. The occurrence of hyperglycemia, as an indicator of T1D development, was assessed by measuring blood glucose levels, using a glucometer (GlucoSure, Apex Biotechnology Group, Hsinchu, Taiwan). All *ex vivo* analyses of the immune response were performed on day 12 after the beginning of T1D induction on 3-6 mice per group.

### 2.4 Cell isolation

#### 2.4.1 Lymph nodes and the pancreatic infiltrates

Cells from the pancreatic lymph nodes were obtained by passing the tissue through a cell strainer (40 μm). After removing the pancreatic lymph nodes, the pancreatic tissue was digested to obtain the pancreatic immune cell infiltrates. The pancreas was cut into small pieces (1–2 mm), washed with Hank’s balanced salt solution (HBSS; Sigma-Aldrich) containing 10% fetal calf serum (FCS; PAA Laboratories, Pasching, Austria), and incubated with Collagenase type V (2 mg/ml, Sigma-Aldrich) dissolved in HBSS + 10% FCS (7.5 ml per pancreas) for 15 min, with constant shaking (200 S/min) at 37°C, after which the samples were vortexed for 20 s. The digests were then passed through a cell strainer and washed with HBSS + 10% FCS. The samples were then layered onto Histopaque^®^-1077 gradient media (Sigma-Aldrich) and after centrifugation (700g, 20 min, no rotor brakes) the mononuclear pancreas-infiltrating cells were collected from the interface. The obtained cells were finally resuspended in RPMI 1640 medium containing 5% FCS, 1% penicillin and streptomycin (all from PAA Laboratories), 2 mM L-glutamine and 25 mM HEPES (Sigma-Aldrich).

#### 2.4.2 Small intestine lamina propria cells

Immune cells from the small intestine (SI) lamina propria were isolated according to the amended protocol by Weigmann et al. (21). Briefly, the SI was removed and cut into pieces (approx. 5 cm long), after which the intestinal content and the Peyer’s patches were removed. The SI was then opened longitudinally, additionally cut into smaller pieces (approx. 1 cm long) and thoroughly washed three times in cold phosphate-buffered saline (PBS; Sigma-Aldrich). Subsequently, the samples were washed with PBS containing 2% FCS and 2.5 mM Dithiothreitol (DTT, Sigma-Aldrich) in an orbital shaker (250 rpm, 15 min) to reduce the presence of mucus. In the next step, the samples were washed three times in PBS containing 2% FCS and 5 mM EDTA (250 rpm, 15 min) to remove the epithelial cells. The SI pieces were gathered and washed in RPMI 1640 supplemented with 10% FCS (250 rpm, 10 min), and resuspended in a solution of Collagenase D (700 µg/ml) and DNase I (0.1 mg/ml) (both from Roche Diagnostics GmbH, Mannheim, Germany) dissolved in RPMI 1640 + 10% FCS. The samples were incubated for 1 h at 37°C in an orbital shaker (350 rpm). After digestion, the tissue was homogenized through a 70 µm cell strainer and washed twice (550g, 5 min). The pellet was resuspended in a 40% Percoll (Cytiva, Marlborough, MA, USA) gradient solution, layered upon 80% Percoll, and centrifuged at 2000 rpm for 20 min without rotor brakes. Lamina propria cells were collected from the interface between 40% and 80% Percoll, washed twice in PBS and resuspended in RPMI 1640 + 5% FCS for further analyses.

#### 2.4.3 Tonsillar cells

Tonsils were taken during the surgery and transported in sterile saline. The tissue was dissected into smaller pieces and passed through the cell strainer to obtain single cell suspension (in PBS + 3% FCS). After centrifugation at 550g for 5 min, pelleted cells were resuspended in RPMI 1640 + 10% FCS and seeded in 96-well plate. Cells were exposed to AGT-5 dissolved in DMSO (0.75 µM) for 48 h, while the control cells were treated with the same volume of DMSO alone.

#### 2.4.4 Dendritic cells

Dendritic cells (DC) were differentiated from bone marrow cells. The femur was isolated and flushed aseptically with RPMI 1640 + 10% FCS. Cells were then centrifuged, erythrocytes lysed with red blood cell lysis buffer and cells resuspended in RPMI 1640 supplemented with 20% FCS, 2 mM L-glutamine and 1 mM Na-pyruvate (Sigma-Aldrich). Obtained cells were cultivated with 20 ng/ml of GM-CSF (Peprotech, London, UK) for 7 days with medium change every second day. Cells were then washed and collected with accutase (Thermo Fisher Scientific, Waltham, MA, USA) treatment and seeded at 50,000 cells/well in a 96-well U-bottom plate (Sarstedt AG & Co. KG, Nümbrecht, Germany). DC were treated with lipopolysaccharide (LPS; 10 ng/ml, Sigma-Aldrich), LPS + AGT-5 (0.75 µM), or DMSO (the same volume as used for AGT-5 dilution), or 1-methyl tryptophan (1MT) in combination with LPS and AGT-5 (0.75 µM) for 24 h.

### 2.5 Flow cytometry

Cell surface molecules were detected on isolated cells from different tissues (pancreas, pancreatic lymph nodes and SI lamina propria). All antibodies were dissolved in Flow Cytometry Staining Buffer (eBioscience, San Diego, CA, USA). The list of used antibodies is displayed in the **Supplementary Tables S1, S3**.

For intracellular cytokine staining, cells were stimulated for 4 h with Cell Stimulation Cocktail (plus protein transport inhibitors) (eBioscience). The cells were then fixed in 2% paraformaldehyde for 15 min at room temperature, permeabilized with Permeabilization buffer (Thermo Fisher Scientific) for 30 min and stained with the fluorescently labelled antibodies (**Supplementary Table S2**).

Treg were detected by Mouse Regulatory T cell Staining Kit (FoxP3) according to the manufacturer’s instructions (eBioscience). RORγt was detected following the same protocol. Each staining was performed for 40 min at 4°C. Isotype-matched controls were included in all experiments (eBioscience). Cell samples were acquired on FACS Aria III (BD Biosciences, Bedford, MA, USA) and analyzed using FlowJo™ 10.10.0 software (BD Life Sciences, Ashland, OR, USA).

### 2.6 Histological analysis

The pancreata were collected, fixed in 4% neutral buffered formalin, and embedded in paraffin. The embedded tissue was cut into 5 μm thick sections with a microtome, with at least 100 μm between sections. The presence of pancreatic islet inflammatory cell infiltrates and the degree of islet cell destruction were evaluated by staining the tissue sections with Mayer’s hematoxylin (Bio-Optica, Milan, Italy) and examined by light microscopy (Leica Microsystems GmbH, Wetzlar, Germany). Insulitis scoring was performed by examining at least 25 islets per pancreas and graded in a blinded fashion: intact islet, without infiltrates; peri-insulitis, with infiltrates in the islet periphery; and insulitis, with infiltrates within the islet. Results are expressed as a percentage of graded islets out of the total number of islets, with three pancreata examined per group.

Insulin expression within the pancreatic islets was evaluated after immunohistochemical staining of the tissue sections with Alexa Fluor^®^ 488-conjugated rabbit anti-mouse insulin antibody (1:400, Cell Signaling Technology, Danvers, MA, USA), with the nuclei counterstained with Hoechst 33342 dye (2 μl/ml, ChemoMetec, Allerød, Denmark). Images were acquired with a Zeiss Axio Imager Z1 florescence microscope (Carl Zeiss Meditec AG, Jena, Germany), at 20× magnification. After converting the images to gray scale, the expression of insulin within the islets was analyzed with the open-source software Fiji (22). Fluorescence intensity was quantified by measuring the mean gray value, which represents the sum of gray values of all pixels in the selected area divided by the total number of pixels. At least 30 islets per pancreas were analyzed, with three pancreata examined per group.

### 2.7 ELISA assay

Insulin concentration in the serum was determined using an Insulin ELISA kit (Millipore, Billerica, MA, USA) according to the manufacturer’s instructions. IL-10 and TNF concentrations in the supernatants of DC cultures were determined by commercial DuoSet^®^ ELISA (R&D Systems, Minneapolis, MN, USA) according to the manufacturer’s instructions. Absorbance was measured by Synergy H1 Hybrid Multi-Mode Reader (BioTek, Swindon, United Kingdom) at 450/570 nm. A standard curve created from the known concentrations of insulin, IL-10 and TNF was used to calculate the concentration values of the tested samples.

### 2.8 Statistical analysis

Data are presented as mean ± SD. The significance of differences between groups was determined by a two-tailed Student’s t-test. Differences are regarded as statistically significant if *p* < 0.05. Statistical analyses were performed using GraphPad Prism 9.0.0 software (GraphPad Software, Inc., La Jolla, CA, USA).

## 3 Results

### 3.1 AGT-5 treatment alleviates clinical and histological parameters of T1D in mice

**Figure 1:**
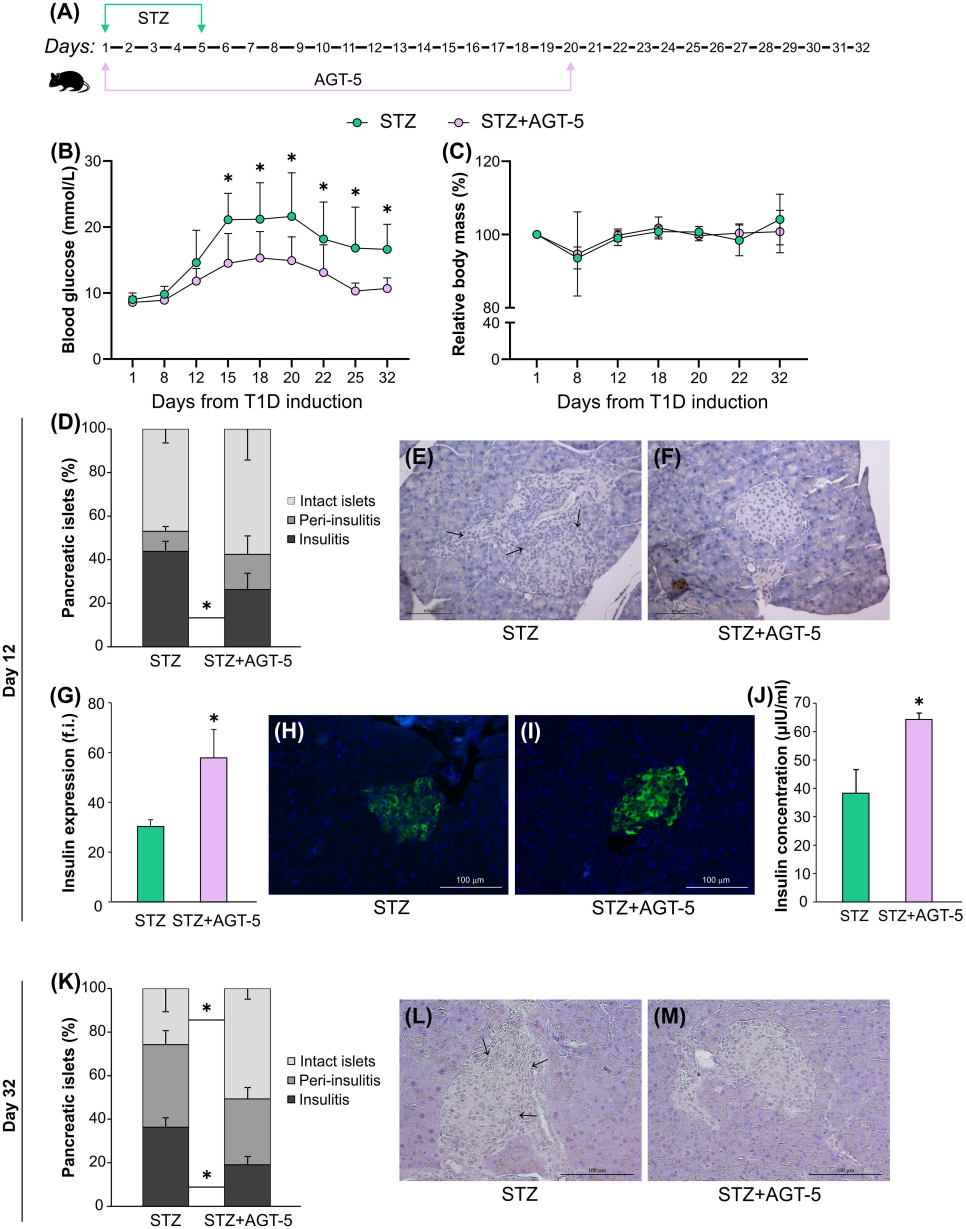
AGT-5 reduced the severity of T1D. **(A)** Diagram of T1D induction by STZ and AGT-5 treatment in C57BL/6 mice. Line graphs show **(B)** blood glucose level (mmol/L) and **(C)** relative body mass in relation to the initial body mass (%). Each group consisted of 8 mice. **(D)** Stacked bar graph shows the proportions of islets without infiltration (intact islets), with immune cells surrounding the islet (peri-insulitis) and with immune cells in the pancreatic islet (insulitis, indicated by arrows) on day 12. Representative images of pancreatic islets from the **(E)** STZ group and **(F)** STZ+AGT-5 group, stained with hematoxylin. **(G)** Histogram shows insulin expression (f.i., fluorescence intensity) in the pancreatic islets. Representative images of pancreatic islets from the **(H)** STZ group and **(I)** STZ+AGT-5 group, stained for visualization of insulin (green) and counterstained with Hoechst 33342 (nucleus – blue). **(J)** Histogram shows serum insulin level (µIU/ml). **(K)** Stacked bar graph shows the proportions of islets without infiltration (intact islets), with immune cells surrounding the islet (peri-insulitis) and with immune cells in the pancreatic islet (insulitis, indicated by arrows) on day 32. Representative images of pancreatic islets from the **(L)** STZ group and **(M)** STZ+AGT-5 group, stained with hematoxylin. Data represent results from one out of three independent experiments. *p<0.05 between the STZ and STZ+AGT-5 groups.

AGT-5 has already been shown to be an effective anti-inflammatory agent as it stimulates proliferation and differentiation of Treg *in vitro* through AHR binding (20). In this study, AGT-5 was administered orally from the first day of T1D induction and treatment continued for 20 days (**Figure 1A**). Herein we confirmed AGT-5’s immunomodulatory properties as it improved T1D clinical signs. In particular, AGT-5 significantly reduced hyperglycemia in T1D mice, and its beneficial effect persisted even after the end of the treatment (on day 20) (**Figure 1B**). Furthermore, AGT-5-treated mice maintained a similar relative body mass to their untreated counterparts throughout the 32-day observation period (**Figure 1C**). The initial body weights in STZ and STZ+AGT-5 groups were similar and ranged between 22.9 g and 26.2 g (24.8 ± 1.4 g) for STZ-treated mice and between 23.4 g and 26.6 g for STZ+AGT-5 treated mice (24.5 ± 1.3 g).

Histological analysis of pancreatic islets harvested on day 12 after T1D induction showed that AGT-5-treated mice had a lower percentage of pancreatic islets with insulitis (26.3 ± 7.4%) compared with the control animals (43.8 ± 4.7%) (**Figures 1D–F**). This was accompanied by the increased production of insulin in the islets (**Figures 1G–I**), as well as by the increased insulin concentration in the serum (**Figure 1J**) of AGT-5-treated animals. The AGT-5 treatment continued to have a positive effect on β-cell protection even after the end of treatment, as judged by the reduced number of infiltrated islets and higher number of intact islets in the pancreata of AGT-treated mice (**Figures 1K–M**), evaluated on day 32.

### 3.2 AGT-5 treatment promotes an anti-inflammatory response in the pancreas and lymph nodes of mice with T1D

**Figure 2:**
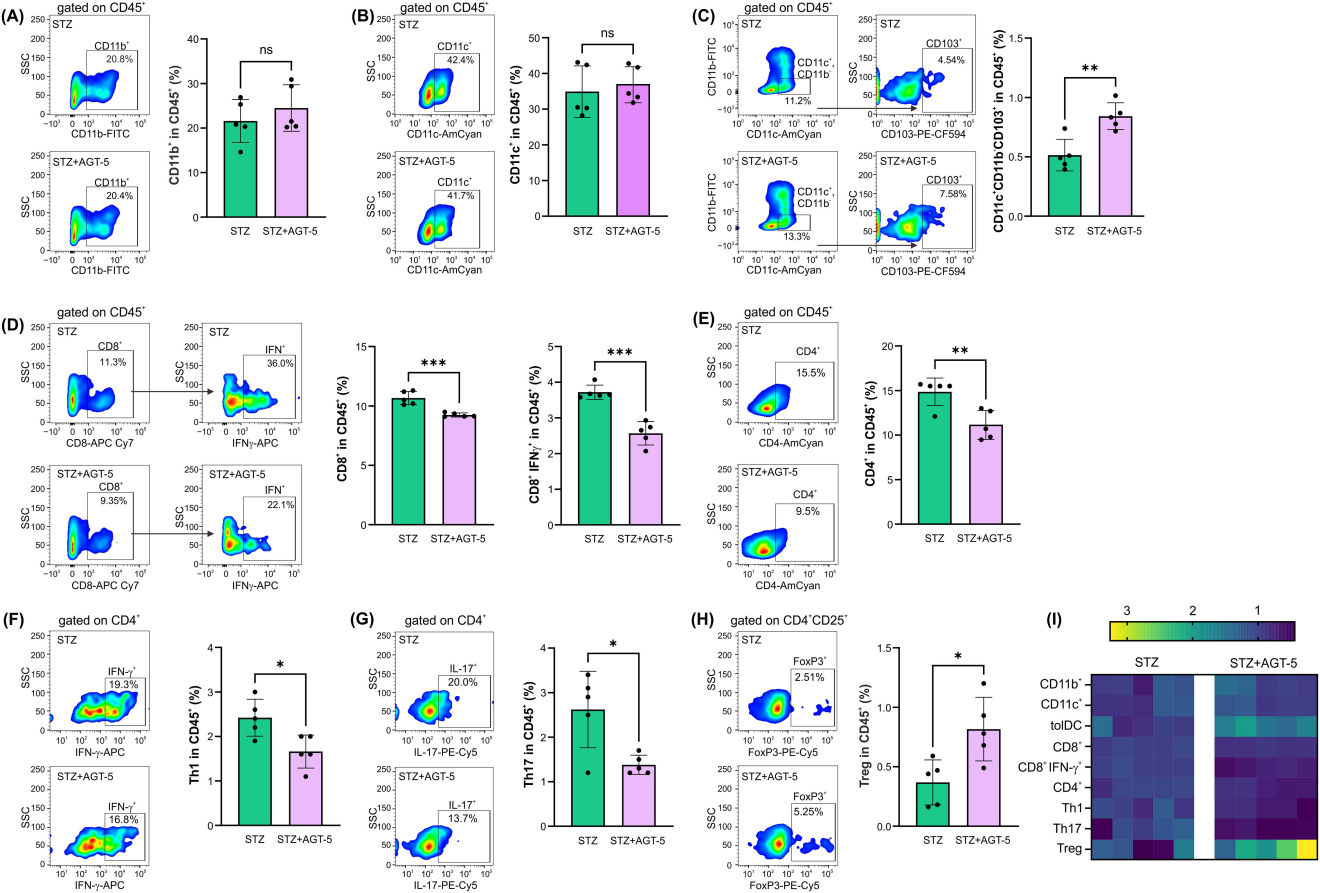
The effect of AGT-5 on immune cells in the pancreas. *Ex vivo* analysis was performed on day 12 after the first STZ injection (5 mice per group). Histograms show the proportions of **(A)** CD11b^+^ cells, **(B)** CD11c^+^ cells, **(C)** tolDC (CD11b^−^CD11c^+^CD103^+^), **(D)** CD8^+^ cells and IFN-γ-producing CD8^+^ cells, **(E)** CD4^+^ cells, **(F)** Th1 (CD4^+^IFN-γ^+^) cells, **(G)** Th17 (CD4^+^IL-17^+^) cells and **(H)** Treg (CD4^+^CD25^+^FoxP3^+^) within CD45^+^ cells, calculated using FlowJo v10.10.0 software. Corresponding flow cytometry plots show the frequencies of examined cell subsets within the indicated parent gates in the representative samples of STZ and STZ+AGT-5 mice. **(I)** Heatmap of the relative proportion of different cell types in STZ and STZ+AGT-5 groups of mice, normalized to the mean value for each cell type of the STZ group. Data represent results from one out of two independent experiments. *p<0.05, **p<0.01, ***p<0.005 between the STZ and STZ+AGT-5 groups. ns, not significant.

Consistent with the decreased infiltration of immune cells in the pancreas of AGT-5-treated mice, flow cytometric analysis of infiltrates on day 12 after the start of disease induction indicated an anti-inflammatory status in this target tissue. The first cells to infiltrate pancreas, after the initial β-cell injury, are macrophages and DC (23). Although the presence of classical antigen-presenting cells (APC), CD11b^+^ or CD11c^+^ was not altered (**Figures 2A, B**), there was a significant increase in the proportion of anti-inflammatory tolDC (CD11c^+^ CD11b^-^ CD103^+^) in AGT-5-treated mice (**Figure 2C**). CD8^+^ cytotoxic lymphocytes, along with the APC, tend to migrate to the pancreatic islets and exert a direct damaging effect on β-cells (23). Treatment with AGT-5 reduced the infiltration of both CD8^+^ cells and IFN-γ-producing CD8^+^ cells (**Figure 2D**). Finally, the infiltration of CD4^+^ helper cells was also reduced (**Figure 2E**) and the balance between the Th cell subsets was shifted towards an anti-inflammatory response, as we observed a reduced population of Th1 and Th17 cells (**Figures 2F, G**) and an increased population of Treg (**Figure 2H**). As can be seen from the heatmap representation of cell occurrence, AGT-5 had the greatest effect on tolDC and Treg (**Figure 2I**). Complementary to the *in vivo* findings, *in vitro* exposure of differentiated (bone marrow-derived) DC to the subsequent treatment with LPS, which induces the full maturation of DC, and AGT-5 (0.75 µM), reduced the proportions of tolDC expressing co-stimulatory molecules CD40, CD80 and CD86 (**Supplementary Figures 5A–C**).

**Figure 3:**
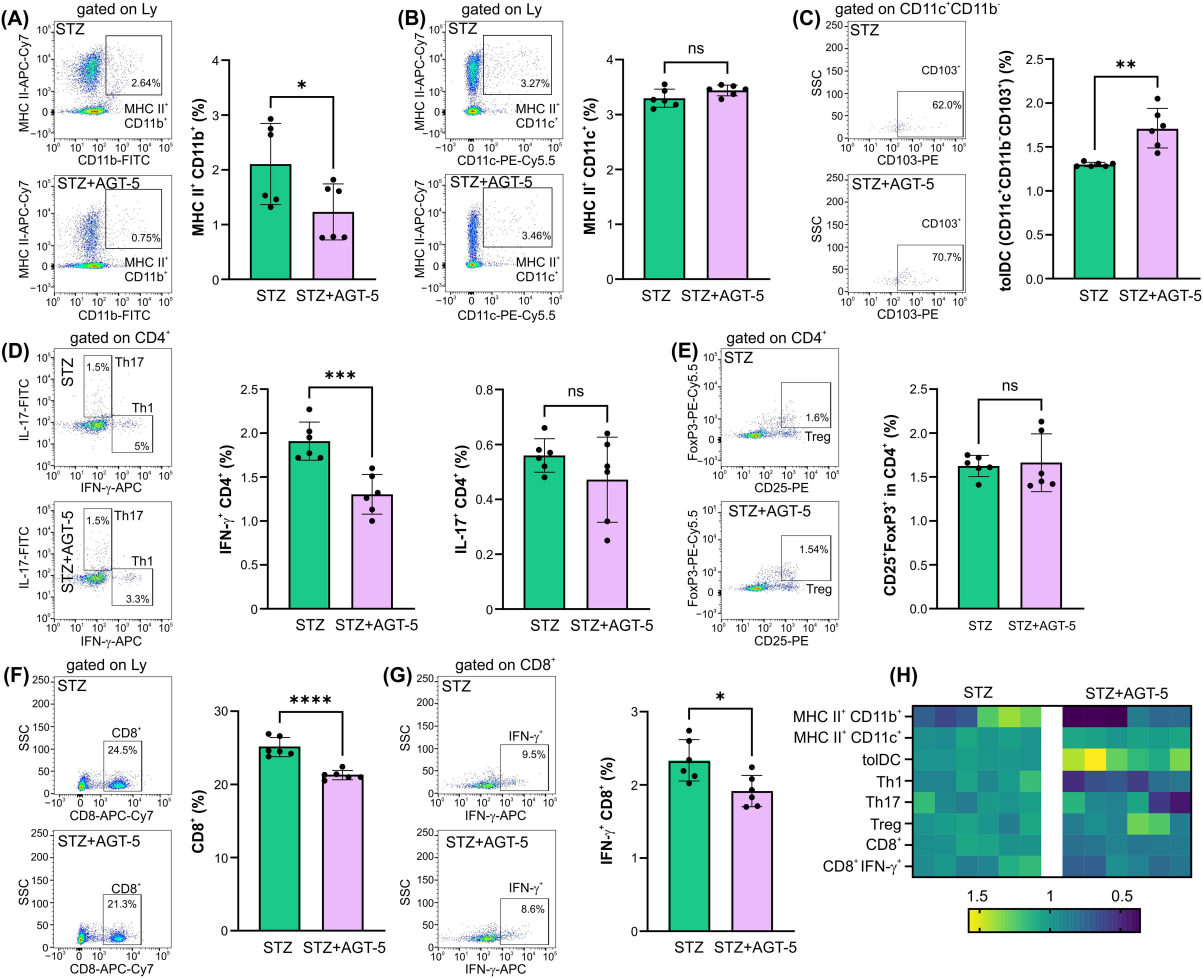
The effect of AGT-5 on cells in the pancreatic lymph nodes. *Ex vivo* analysis was performed on day 12 after the first STZ injection (6 mice per group). Histograms show the proportions of **(A)** MHC II^+^CD11b^+^ cells, **(B)** MHC II^+^CD11c^+^ cells, **(C)** tolDC (CD11b^−^CD11c^+^CD103^+^), **(D)** Th1 (CD4^+^IFN-γ^+^) and Th17 (CD4^+^IL-17^+^) cells, **(F)** CD8^+^ and **(G)** CD8^+^IFN-γ^+^ cells within lymph node lymphocytes, and **(E)** Treg (CD25^+^FoxP3^+^) within CD4^+^ lymph node cells, calculated using FlowJo v10.10.0 software. Corresponding flow cytometry plots show the frequencies of examined cell subsets within the indicated parent gates in the representative samples of STZ and STZ+AGT-5 mice. **(H)** Heatmap shows the relative proportions of different cell types in STZ and STZ+AGT-5 groups of mice normalized to the mean value for each cell type of the STZ group. Data represent results from one out of two independent experiments. *p<0.05, **p<0.01, ***p<0.005, ****p<0.001 between the STZ and STZ+AGT-5 groups. ns, not significant.

The distribution of immune cell populations in the draining pancreatic lymph nodes was similar to the one observed in the pancreas. AGT-5 efficiently down-regulated the proportion of MHC II^+^ CD11b^+^ APC (**Figure 3A**) and increased the proportion of tolDC in the pancreatic lymph nodes (**Figure 3C**), with no observed effect on MHC II^+^ CD11c^+^ cells (**Figure 3B**). As for the CD4^+^ cell population, AGT-5 reduced the frequency of pro-inflammatory Th1 cells, while the proportions of Th17 cells and Treg remained unchanged (**Figures 3D, E**). Importantly, AGT-5 reduced the proportion of CD8^+^ cells, including those that produce IFN-γ (**Figures 3F, G**). Analysis of cell composition in the lymph nodes revealed AGT-5’s significant impact on CD11b^+^ cells and tolDC (**Figure 3H**).

### 3.3 AGT-5 treatment promotes an anti-inflammatory response in the lamina propria of mice with T1D

**Figure 4:**
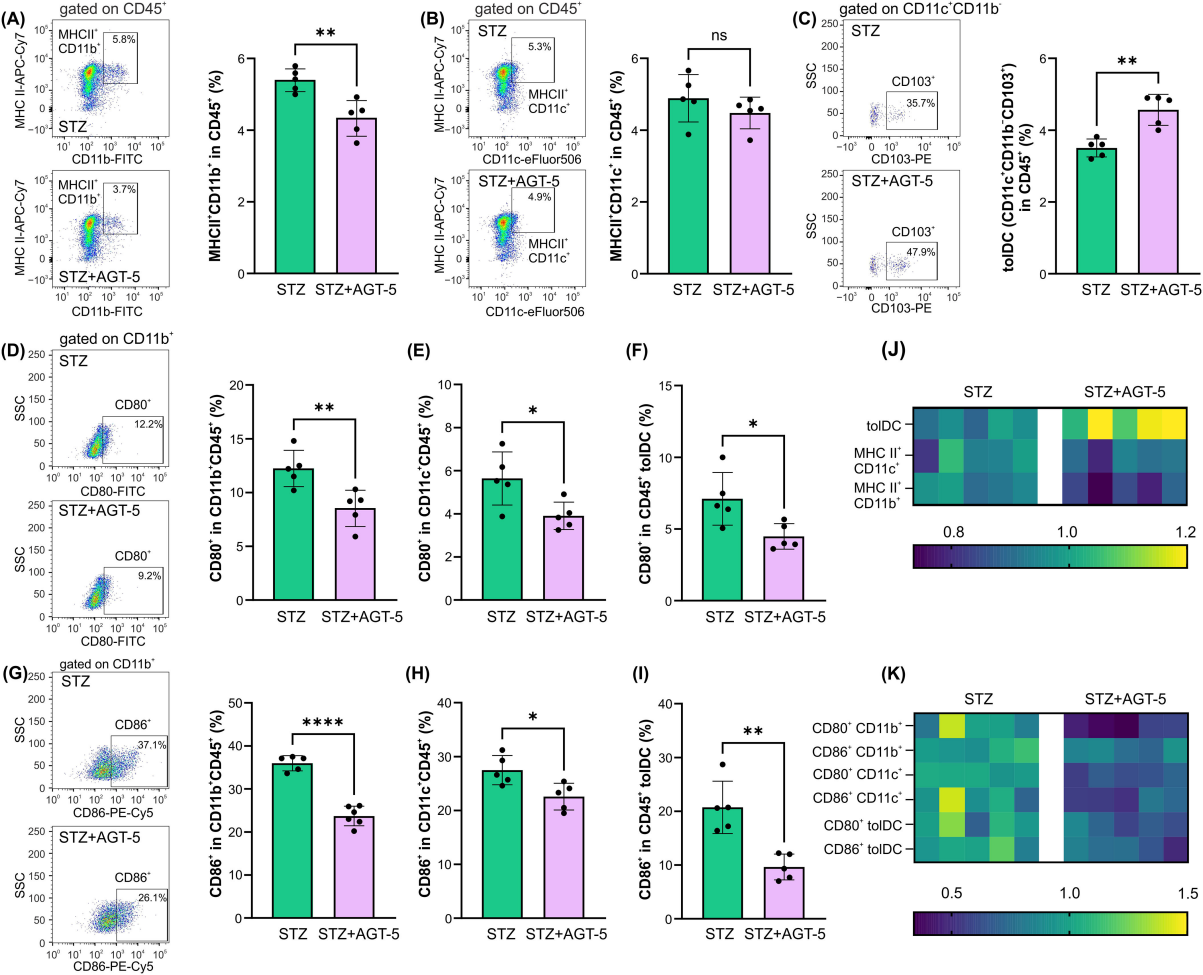
The effect of AGT-5 on innate immune cells in the SI lamina propria. *Ex vivo* analysis was performed on day 12 after the first STZ injection (5 mice per group). Histograms show the proportions of **(A)** MHC II^+^CD11b^+^ cells, **(B)** MHC II^+^CD11c^+^ cells, **(C)** tolDC (CD11b^−^CD11c^+^CD103^+^) within CD45^+^ cells, and CD80^+^ cells within **(D)** CD11b^+^CD45^+^ cells, **(E)** CD11c^+^CD45^+^ cells, **(F)** CD45^+^ tolDC, or CD86^+^ cells within **(G)** CD11b^+^CD45^+^, **(H)** CD11c^+^CD45^+^ cells and **(I)** CD45^+^ tolDC, calculated using FlowJo v10.10.0 software. Corresponding flow cytometry plots show the frequencies of examined cell subsets within the indicated parent gates in the representative samples of STZ and STZ+AGT-5 mice. **(J, K)** Heatmaps show the relative proportions of different cell types in STZ and STZ+AGT-5 groups of mice normalized to the mean value for each cell type of the STZ group. Data represent results from one out of two independent experiments. *p<0.05, **p<0.01, ****p<0.001 between the STZ and STZ+AGT-5 groups. ns, not significant.

As AGT-5 was administered orally and the predominant AHR-expressing immune cells reside within the SI lamina propria (24), we next performed e*x vivo* analysis of cells within this tissue on day 12 after the initiation of T1D induction. The results demonstrated AGT-5’s predominant effect on tolDC, ILC3, and Treg. Specifically, AGT-5 treatment down-regulated the frequency of MHC II-expressing CD11b^+^ APC (**Figure 4A**), did not change the proportion of MHC II-expressing CD11c^+^ APC (**Figure 4B**) and upregulated the proportion of tolDC (**Figure 4C**). The proportions of CD11b^+^ and CD11c^+^ cells and tolDC expressing the co-stimulatory molecules CD80 and CD86 were reduced upon AGT-5 treatment (**Figures 4D–I**). Overall, the major differences between the treated and control mice were observed in the tolDC compartment (**Figures 4J, K**).

**Figure 5:**
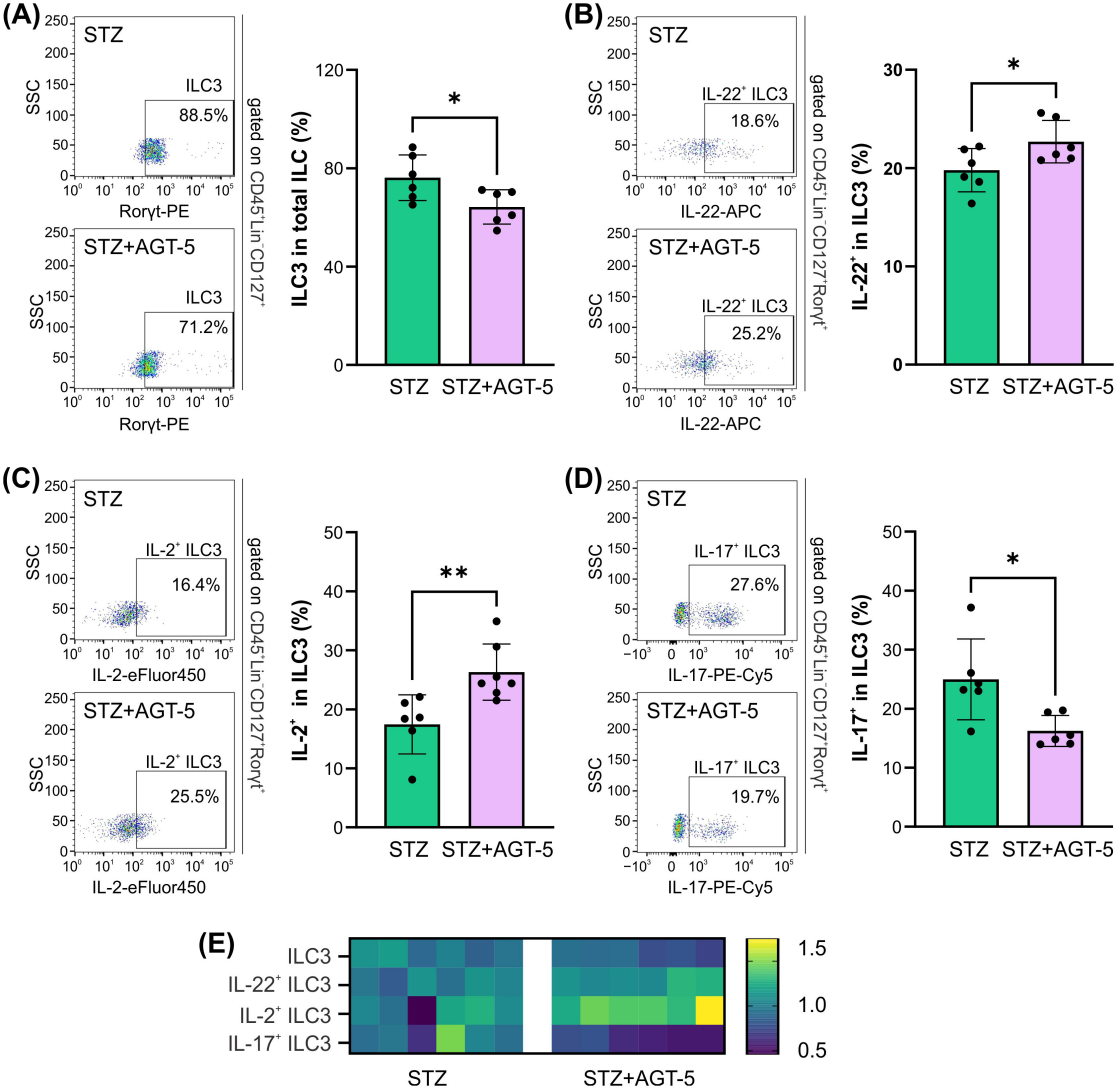
The effect of AGT-5 on ILC3 in the SI lamina propria. Histograms depict the proportions of **(A)** ILC3 (RORγt^+^) within total ILC (CD45^+^Lin^−^CD127^+^), **(B)** IL-22-producing cells within ILC3, **(C)** IL-2-producing cells within ILC3 and **(D)** IL-17-producing cells within ILC3, calculated using FlowJo v10.10.0 software (6 mice per group, day 12 after the initiation of the experiment). Corresponding flow cytometry plots show the frequencies of examined cell subsets within the indicated parent gates in the representative samples of STZ and STZ+AGT-5 mice. **(E)** Heatmap shows the relative proportions of different cell types in STZ and STZ+AGT-5 groups of mice normalized to the mean value for each cell type of the STZ group. *p<0.05, **p<0.01 between the STZ and STZ+AGT-5 groups.

Another important population of cells that reside in gut-associated lymphoid tissue (GALT) and is dependent on AHR expression is ILC3 population (25). Although ILC3 proportions were reduced in AGT-5-treated mice in comparison to control diabetic mice and (**Figure 5A**), the frequency of IL-22^+^ and IL-2^+^ cells in ILC3 were upregulated after AGT-5 treatment (**Figures 5B, C**). In contrast, AGT-5 down-regulated the ILC3 subpopulation that produces IL-17 (**Figure 5D**). The heatmap illustrates the primary impact of AGT-5 on IL-2 production in ILC3 (**Figure 5E**).

**Figure 6:**
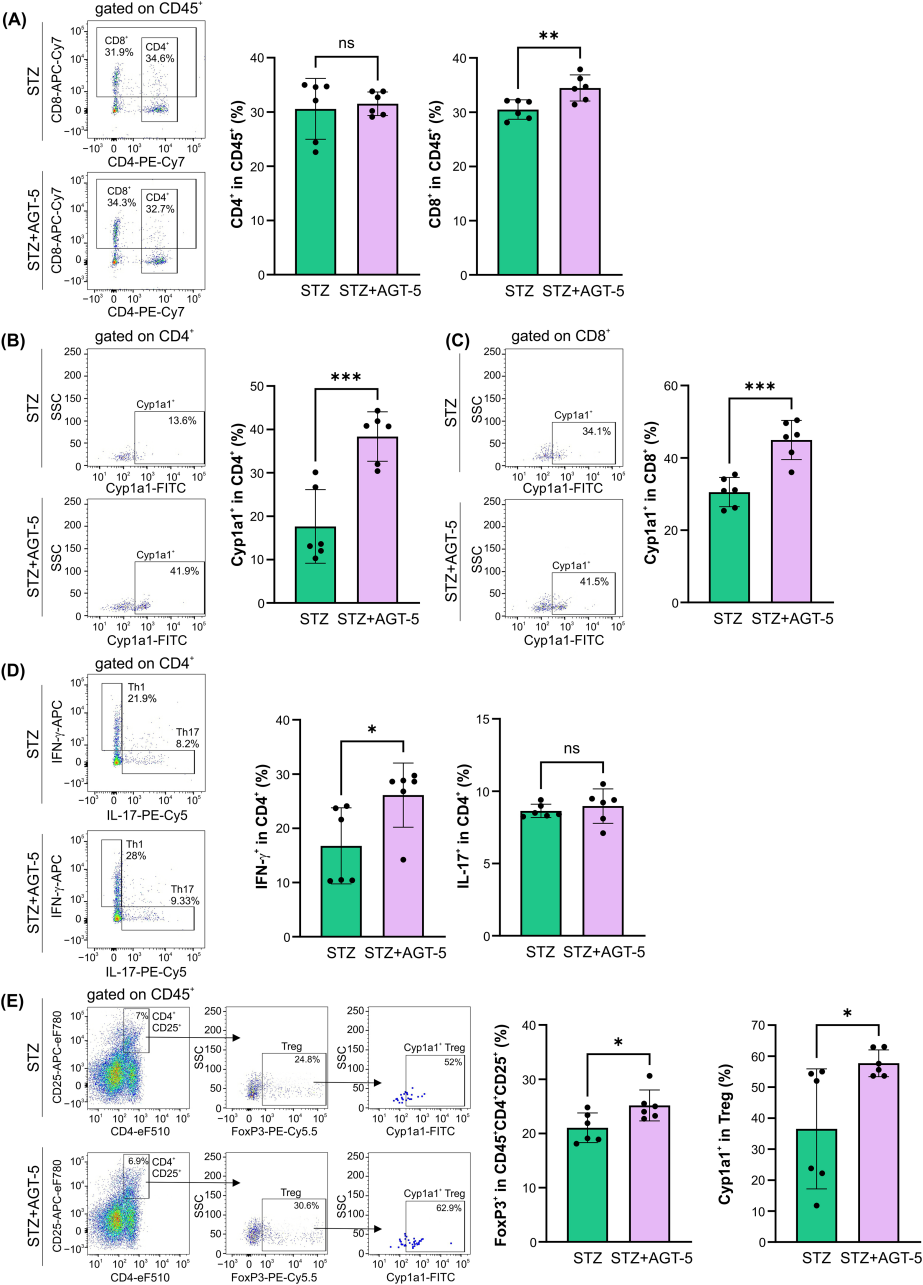
The effect of AGT-5 on the adaptive immune cells in the SI lamina propria. *Ex vivo* analysis was performed on day 12 after the first STZ injection (6 mice per group). Histograms show the proportions of **(A)** CD4^+^ and CD8^+^ cells within CD45^+^ cells, **(B, C)** Cyp1a1^+^ cells within **(B)** CD4^+^ and **(C)** CD8^+^ cells, **(D)** Th1 (IFN-γ^+^) and Th17 (IL-17^+^) cells within CD4^+^ cells, and **(E)** FoxP3^+^ Treg within CD4^+^CD25^+^CD45^+^ cells and Cyp1a1^+^ cells among them, calculated using FlowJo v10.10.0 software. Corresponding flow cytometry plots show the frequencies of examined cell subsets within the indicated parent gates in the representative samples of STZ and STZ+AGT-5 mice. Data represent results from one out of two independent experiments. *p<0.05, **p<0.01, ***p<0.005 between the STZ and STZ+AGT-5 groups. ns, not significant.

AGT-5 influenced the adaptive immune response in the SI lamina propria. While AGT-5 did not affect the proportion of CD4^+^ cells, it increased the proportion of CD8^+^ lymphocytes (**Figure 6A**). As a confirmation of its AHR-related effects, AGT-5 significantly upregulated the proportion of Cyp1a1-expressing CD4^+^ and CD8^+^ lymphocytes (**Figures 6B, C**). Also, AGT-5 upregulated the frequency of Th1 cells, but exerted no effect on Th17 cell subset (**Figure 6D**). In addition to enhancing the proportion of Treg within the SI lamina propria, AGT-5 also increased the frequency of Cyp1a1-expressing Treg, confirming that the observed immunomodulatory effects are mediated through AHR (**Figure 6E**). Of note, a similar stimulatory effect of AGT-5 was observed *in vitro* on Cyp1a1-expressing DC (**Supplementary Figure S6**).

### 3.4 AGT-5 treatment stimulates tolDC and Treg functions in the pancreas and lamina propria of T1D mice

**Figure 7:**
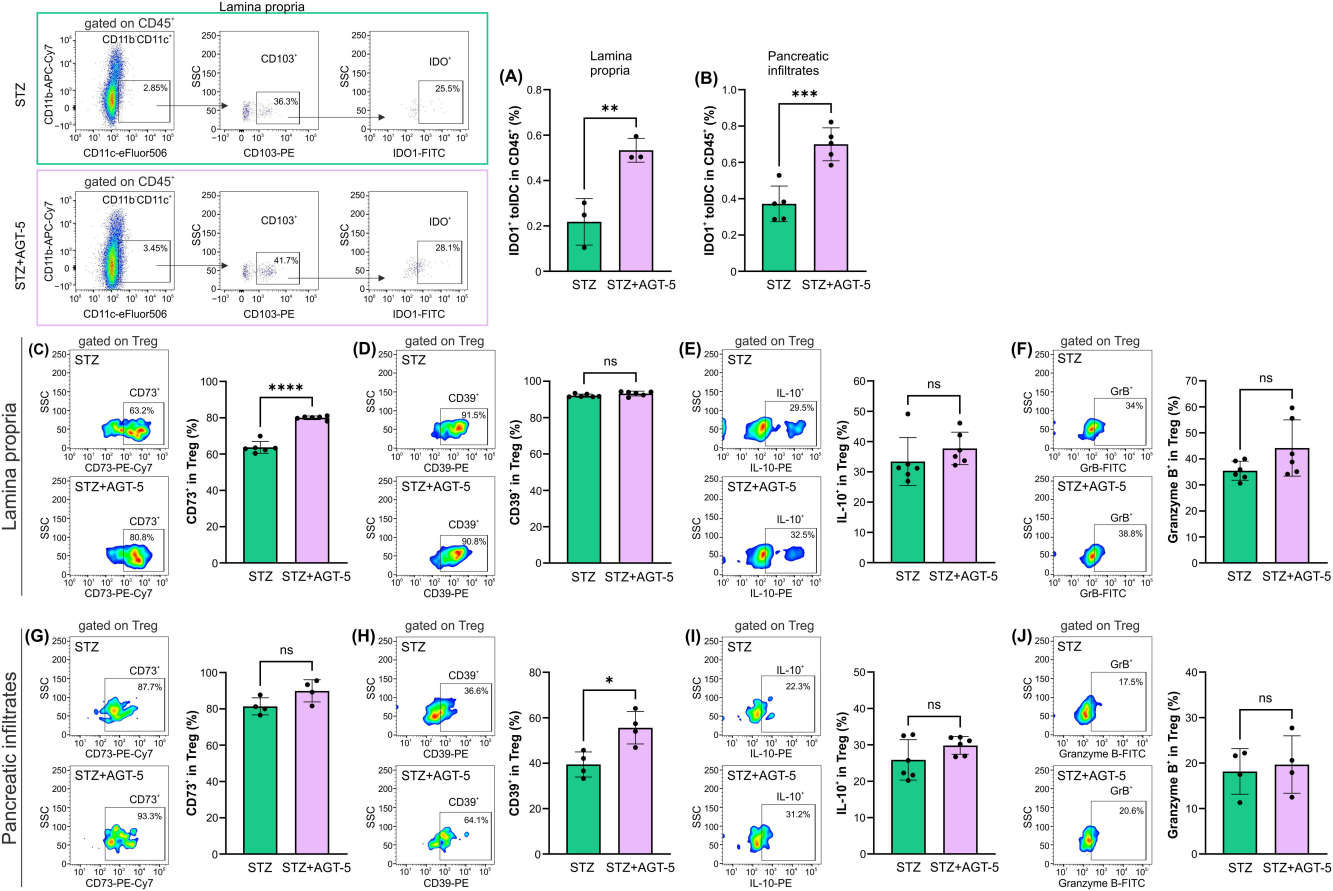
The effect of AGT-5 on tolDC and Treg populations in SI lamina propria and pancreatic infiltrates. *Ex vivo* analysis was performed on day 12 after the first STZ injection (3-6 mice per group). Histograms show the proportions of **(A, B)** IDO1^+^ tolDC within CD45^+^ cells in the **(A)** lamina propria and **(B)** pancreatic infiltrates; **(C, G)** CD73^+^ cells, **(D, H)** CD39^+^ cells, **(E, I)** IL-10^+^ cells and **(F, J)** Granzyme B^+^ cells within Treg in the **(C–F)** lamina propria and **(G–J)** pancreatic infiltrates. Corresponding flow cytometry plots show the frequencies of examined cell subsets within the indicated parent gates in the representative samples of STZ and STZ+AGT-5 mice. The frequencies of IDO1^+^ tolDC within CD45^+^ cells were calculated using FlowJo v10.10.0 software. Data represent results from one out of two independent experiments. *p<0.05, **p<0.01, ***p<0.005, ****p<0.001 between the STZ and STZ+AGT-5 groups. ns, not significant.

As AHR activation can directly influence indoleamine 2,3-dioxigenase (IDO1) enzyme expression (26), we have investigated the presence of IDO1^+^ tolDC within SI lamina propria and pancreas. Within both tissues, AGT-5 increased the proportion of IDO1^+^ tolDC, indicating their enhanced anti-inflammatory properties and a strong capacity to induce Treg (**Figures 7A, B**). The effect of AGT-5 on IDO-1 was corroborated by *in vitro* results on mouse bone marrow-derived DC, as AGT-5 exposure enhanced the proportion of IDO1^+^ DC (**Supplementary Figure S7A**). IDO1 may represent a primary target molecule for AGT-5 as the presence of IDO1 inhibitor 1-methyl tryptophan (1MT) prevented the down-regulation of activation markers CD40, CD80 and CD86 (**Supplementary Figures S7B–D**). Additionally, the reduced production of TNF imposed by AGT-5 was increased in the presence of 1MT, while AGT-5-provoked upregulation of IL-10 was absent in 1MT-treated DC, determined by ELISA (**Supplementary Figures S7E, F**). Alongside the effect on DC, Treg immunosuppressive function in SI lamina propria was also increased after AGT-5 treatment as evidenced by the higher proportion of CD73^+^ Treg (**Figure 7C**). However, AGT-5 did not change the proportions of IL-10^+^, CD39^+^ or granzyme B^+^ Treg in this tissue (**Figures 7D–F**). Within the pancreas, AGT-5 increased the proportion of CD39^+^ cells (**Figure 7H**), and had no effect on the frequencies IL-10^+^, CD73^+^ or granzyme B^+^ Treg (**Figures 7G, I, J**). These results suggest that AHR activation by AGT-5 enhances the expression of ATP-degrading enzymes (CD39 and CD73) (27).

### 3.5 The effect of AGT-5 *in vitro* on human DC and ILC3

**Figure 8:**
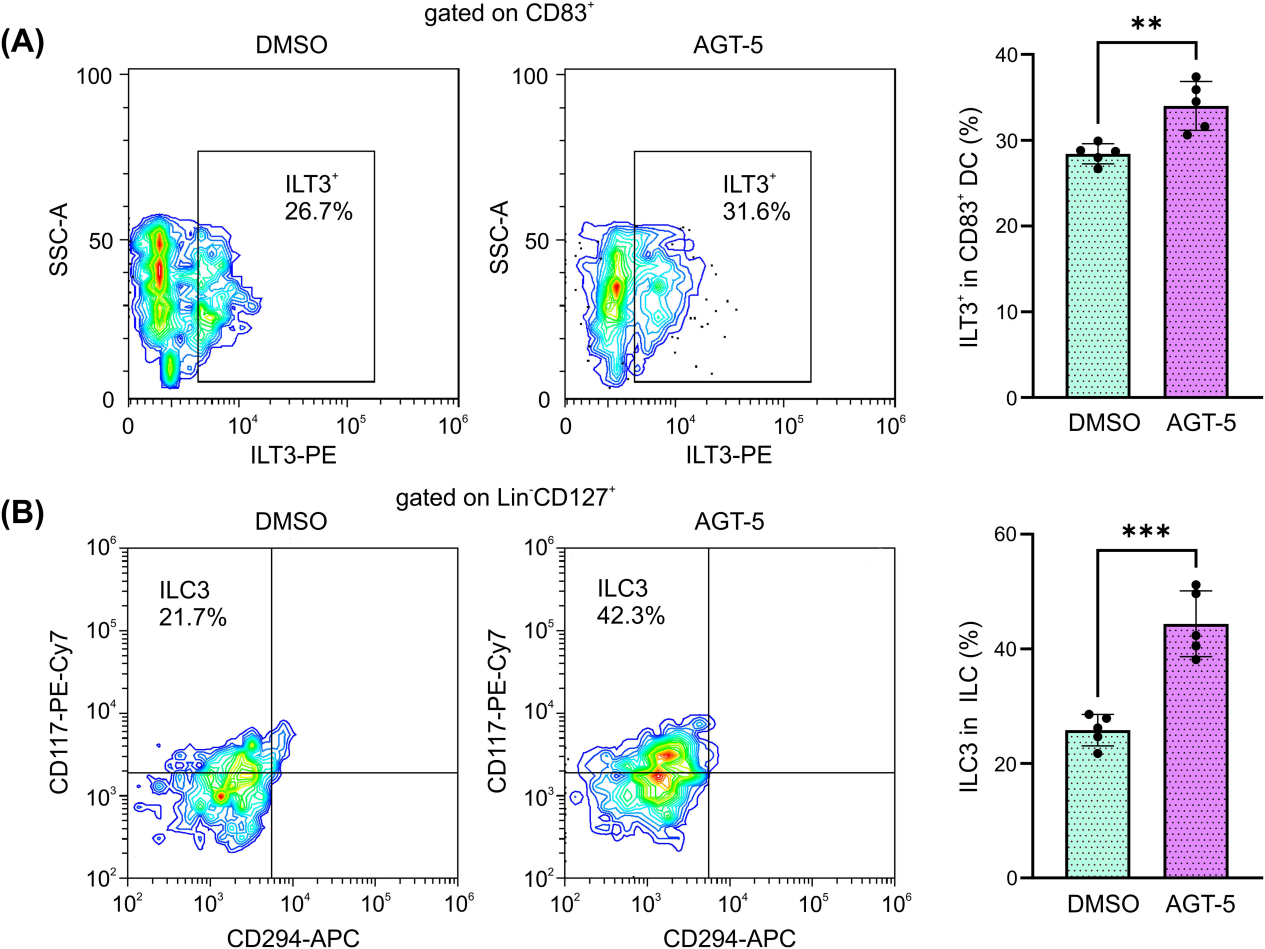
AGT-5 increases the proportion of immunosuppressive DC and ILC3 that originate from human tonsils. Tonsil cell suspension was incubated with AGT-5 (0.75 µM) for 48 h, and flow cytometry was used to detect **(A)** ILT3^+^ DC and **(B)** Lin^−^CD127^+^CRTH2(CD294)^−^CD117^+^ ILC3. Histograms show the proportions of **(A)** ILT3^+^ cells within CD83^+^ DC, and **(B)** CRTH2^−^CD117^+^ ILC3 within total ILC. Representative flow cytometry plots are shown. Representative experiment out of two performed is displayed. **p<0.01, ***p<0.005 between the DMSO-treated and AGT-5-treated cells.

AGT-5 has previously been shown to upregulate human Treg proliferation *in vitro* (20). Here it is shown that *in vitro* exposure of human tonsil cells to AGT-5 (0.75 µM) increased the presence of immunosuppressive DC that express inhibitory molecule ILT3 (**Figure 8A**), suggesting that AGT-5 exerts similar effects on mouse and human DC. At the same time, AGT-5 increased the proportion of ILC3 within the whole ILC population in the tonsils (**Figure 8B**). The gating strategy for ILC3 is displayed in **Supplementary Figure S8**.

## 4 Discussion

Type 1 diabetes (T1D) presents a significant unmet clinical need despite advancements in treatment options. Novel approaches targeting the autoimmune component of T1D and aiming to restore immune tolerance or preserve β-cell function are urgently needed to improve outcomes and quality of life for patients with this chronic condition. Herein, we explored the potential to restore immune tolerance via activating the AHR. Along these lines, we evaluated the effect of the oral administration of AGT-5, an AHR-activating compound with fluorescent properties that we recently developed. AGT-5 attenuated the severity of T1D in a murine STZ model of autoimmune diabetes through the blockade of immune cell infiltration into the pancreatic islets, which enabled sufficient insulin production. These beneficial effects on the clinical course of T1D occurred as a consequence of suppression of inflammation, mediated by AHR-driven upregulation of tolDC and Treg compartments that exerted an immunosuppressive function in the GALT and pancreas.

The AHR is a ligand-activated transcription factor that is expressed by many immune cells and can be activated by various environmental factors, dietary components, microorganisms and metabolites. For example, AHR activation by its high-affinity ligand TCDD *in vivo* suppresses the development of experimental autoimmune encephalomyelitis (EAE), experimental autoimmune uveoretinitis, and spontaneous autoimmune diabetes (15, 28, 29). Although it has strong immunomodulatory properties, TCDD is extremely toxic even in picomolar concentrations and therefore cannot be exploited for human use (30). This feature of TCDD is shared by numerous other AHR ligands. However, several AHR ligands isolated from natural sources (e.g. cruciferous plants) or chemically synthetized ligands (31) exert both low toxicity and a capacity for immunomodulation. AGT-5 belongs to the FluoAHRL family of recently synthetized compounds demonstrating AHR agonistic features and NIR emission. *In vitro* data suggest that AGT-5 preferentially stimulates Treg proliferation and differentiation, and increases Treg function (20). In addition to being a potent pro-Treg driver, AGT-5 showed no signs of toxicity at micromolar concentrations in zebrafish embryos or when administered to mouse macrophages *in vitro* (20). Therefore, this compound was selected as a suitable candidate for the modulation of the inflammatory autoimmune process during T1D development in the STZ animal model.

As expected, according to its effects *in vitro*, AGT-5 successfully attenuated T1D clinical symptoms in mice through interference with both the innate and adaptive immune responses in the pancreas, the draining lymph nodes and SI lamina propria. Initial events related to the pathogenesis of T1D in the STZ model encompass APC infiltrating the pancreas. These cells aim to repair the damage imposed by STZ, but also serve to present engulfed autoantigens and thereby initiate the autoimmune response. AGT-5 successfully reduced the relative number of classical MHC II^+^ CD11b^+^ APC in pancreatic draining lymph nodes, although it did not affect the prevalence of CD11b^+^ APC within the pancreatic infiltrates. In addition to interference with the proportions of macrophages and DC, AGT-5 down-regulated the frequency of cells expressing co-stimulatory molecules CD40, CD80 and CD86 within CD11b^+^, CD1c^+^ and tolDC suggesting the reduced ability of APC to trigger T cell response. Cells that counteract classical APC are tolDC, and their abundance was consistently increased along the SI lamina propria-pancreatic lymph node-pancreas axis after the treatment with AGT-5. The potential for increasing the tolDC response is not uniquely attributed to AGT-5, as several other AHR ligands have shown similar properties. For example, AHR activation with the AHR ligand ITE (16, 32) or with the synthetic agonist laquinimod induces a tolerogenic phenotype in mouse and human DC (33–35). Also, nanoparticles loaded with ITE exert similar pro-tolerogenic effects and suppress the development of EAE (32).

The main feature of T1D is the imbalance between CD4^+^ or CD8^+^ Teff and the FoxP3^+^CD4^+^ Treg which leads to the destruction of pancreatic β-cells, ultimately causing insulin deficiency (4). According to our data, AGT-5 successfully attenuated the severity of T1D by favoring immunosuppressive Treg at the expense of pathogenic Th1 and Th17 cells in the pancreatic infiltrates. The interference with the gut-associated immune response is extremely important for the modulation of T1D as it has been demonstrated that patients with T1D exhibit subclinical intestinal immune activation that may be an indirect proof of the relation between gut inflammation and pancreatic islet-directed autoimmunity (36). Moreover, the activation of insulin-specific T cells can occur in the GALT and their presence was confirmed in Peyer’s patches and mesenteric lymph nodes almost at the same frequency as in the pancreatic lymph nodes (37). In line with these findings, a recent study demonstrated that the disruption of gut barrier continuity leads to the activation of islet-reactive T cells in the intestine, ultimately contributing to the development of autoimmune diabetes (38). In our previous study it was found that AGT-5 can diffuse into the SI lamina propria where it can bind to AHR in immune cells (20). This study confirms that AGT-5 activates AHR in lamina propria, as higher proportions of CD4^+^, CD8^+^ cells and Treg that expressed Cyp1a1 (the primary target gene for AHR) were detected. AGT-5 can actually stimulate *de novo* differentiation of Treg as it was shown that AHR enhances the expression of *Foxp3* mRNA (39). According to the results by Guo et al. (40), targeting and possible manipulation of Treg in the intestinal lamina propria may be beneficial for achieving tolerance and suppressing the autoimmune response. Therefore, AGT-5 can directly, through AHR-driven events, stimulate Treg within the SI lamina propria and this anti-inflammatory environment can be carried over further to the target tissue.

In addition to the direct activation of Treg, AGT-5 can also stimulate Treg through modulation of other cells. For example, ILC3 express high levels of AHR and their differentiation and viability is completely dependent upon AHR (25, 41, 42). In this study, AGT-5 down-regulated the proportion of IL-17^+^ within ILC3 (responsible for anti-microbial response), and increased the frequency of ILC3 that produce IL-2. As IL-2 is a well-recognized Treg growth factor, it would be reasonable to assume that IL-2^+^ ILC3 participated in the upregulation of Treg after AGT-5 treatment. Indeed, there are studies suggesting that IL-2, derived from ILC3, is responsible for proper Treg activation in the lamina propria (43, 44). Also, ILC3 importance for T1D development is reflected by the reduced numbers of IL-2^+^ ILC3 in the lamina propria that preceded the occurrence of insulitis in NOD mice and the appearance of STZ-induced T1D (44). In addition, AGT-5 increased the number of ILC3 that produced IL-22 and thereby may have improved epithelial barrier stability that is usually disturbed during T1D in both mice and humans (44–47).

One of the possible indirect Treg-stimulatory actions of AGT-5 can be exerted through the AGT-5-mediated influence on APC. More specifically, Treg can be activated through the action of tolDC. Activation of AHR was shown to induce the expression of tolerogenic markers of DC – enzymes IDO1 and 2 (26), which, through depriving tryptophan and increasing kynurenine production, promote FoxP3^+^ Treg differentiation. This is in line with our data, as AGT-5 increased the proportion of IDO1^+^ tolDC in both SI lamina propria and the pancreas. *In vitro* results on bone marrow-derived DC support *ex vivo* data since AGT-5 efficiently blocked differentiation of DC into the fully mature APC through down-regulation of co-stimulatory molecules CD80, CD86 and CD40. IDO1 is probably responsible for these effects, as the inhibition of IDO1 by 1MT abrogated the AGT-5-mediated reduction of co-stimulatory molecules in DC.

AGT-5 changes the proportion of Treg, but also affects their function. Treg function is exerted through secretion of IL-10, ATP depletion, expression of inhibitory (CTLA-4, PD-1) or cytotoxic (granzyme B) molecules that inhibit Teff (4). Seemingly, AGT-5 did not stimulate IL-10 production in the T1D setting, which is in contrast to the results obtained from healthy murine SI lamina propria cells (20). In this inflammatory setting, AGT-5 predominantly influenced the ATP depletion machinery in Treg as it increased the proportion of CD73^+^ Treg in the pancreas and CD39^+^ Treg in the SI lamina propria. It is reasonable to assume that these are AHR-related events as it was shown that AHR drives the expression of the ectoenzyme CD39, which cooperates with CD73 to deplete the pro-inflammatory extracellular ATP and catalyze its conversion into anti-inflammatory adenosine (27).

While the present study suggests potential cellular and molecular targets of AGT-5 in the treatment of T1D, it comes with several limitations. First, to identify specific molecular targets and determine precise mechanism of AGT-5 action, RNA sequencing may be used. Second, this study does not cover the possible effect of AGT-5 on the phenomenon termed “Treg resistance”. This represents the lack of sensitivity of Teff towards Treg-mediated suppression, and it has been described in multiple sclerosis, rheumatoid arthritis and T1D patients (48–50). Finally, for the purpose of conducting translational research, pharmacokinetic status of AGT-5 should be explored in future studies.

**Figure 9:**
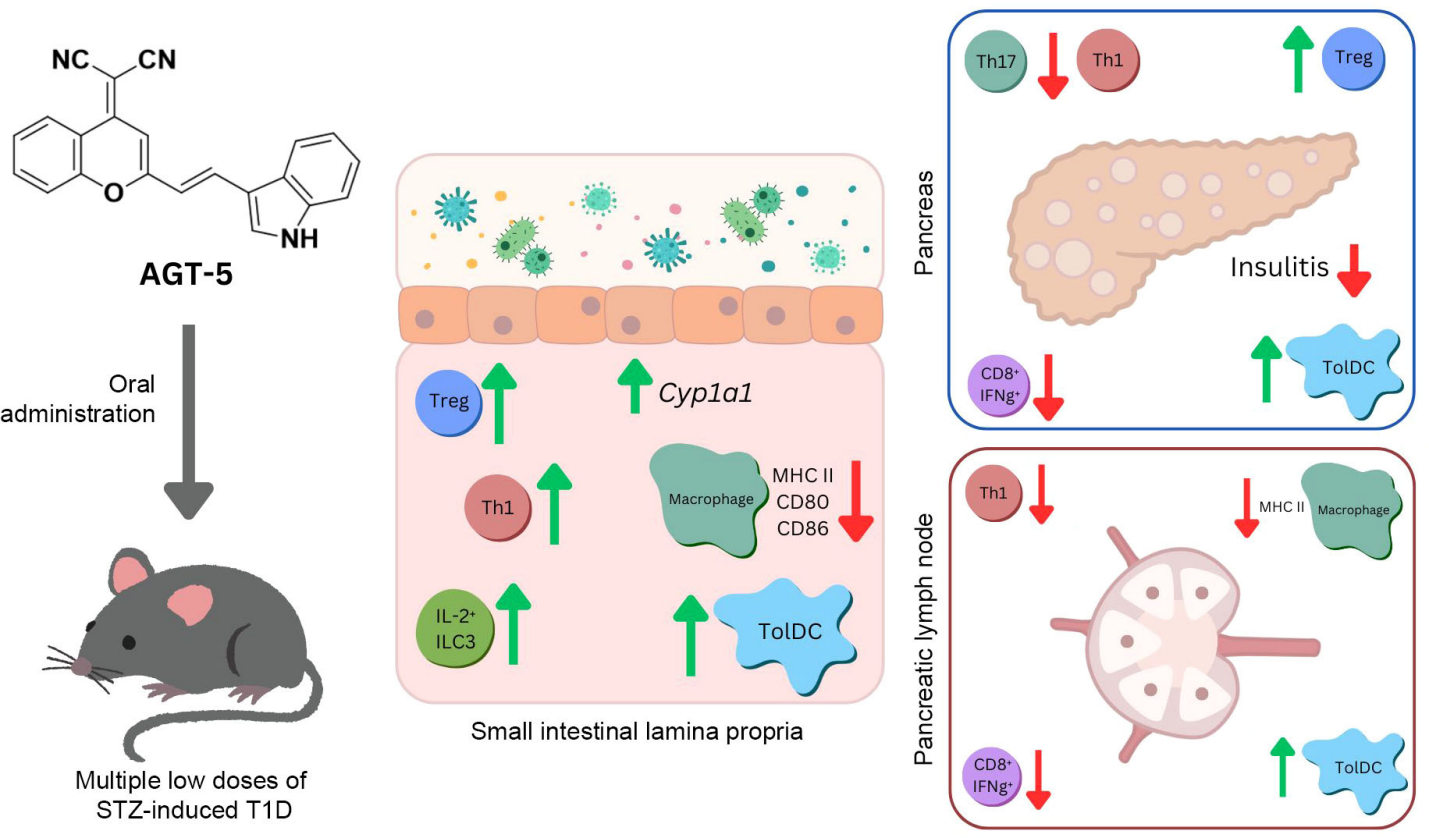
Schematic presentation of AGT-5 impact on T1D pathogenesis.

In conclusion, our study demonstrates that the novel nontoxic fluorescent AHR ligand AGT-5, when administered orally, acts on tolDC, ILC3 and T cells to promote the induction of functional FoxP3^+^ Treg in the GALT and pancreatic islets, thereby inhibiting insulitis and preserving insulin production (**Figure 9**). In addition to its potential as an immunomodulatory therapeutic, AGT-5, due to its fluorescent properties, can be used for *in vivo* imaging. This allows for the visualization of AHR activation and distribution within living organisms. The fluorescent capability of AGT-5 can facilitate and accelerate research in targeting AHR-related pathways for potential therapeutic interventions.

## Data Availability

The original contributions presented in the study are included in the article/**Supplementary Material**. Further inquiries can be directed to the corresponding author.

## References

[1] Daneman D. Type 1 diabetes. Lancet. (2006) 367:847–58. doi: 10.1016/S0140-6736(06)68341-4 16530579

[2] Lehuen A Diana J Zaccone P Cooke A. Immune cell crosstalk in type 1 diabetes. Nat Rev Immunol. (2010) 10:501–13. doi: 10.1038/nri2787 20577267

[3] Coppieters KT Dotta F Amirian N Campbell PD Kay TW Atkinson MA. Demonstration of islet-autoreactive CD8 T cells in insulitic lesions from recent onset and long-term type 1 diabetes patients. J Exp Med. (2012) 209:51–60. doi: 10.1084/jem.20111187 22213807 PMC3260877

[4] Yu H Paiva R Flavell RA. Harnessing the power of regulatory T-cells to control autoimmune diabetes: overview and perspective. Immunology. (2018) 153:161–70. doi: 10.1111/imm.12867 PMC5765377 29155454

[5] Sakaguchi S Sakaguchi N Asano M Itoh M Toda M. Immunologic self-tolerance maintained by activated T cells expressing IL-2 receptor alpha-chains (CD25). Breakdown of a single mechanism of self-tolerance causes various autoimmune diseases. J Immunol. (1995) 155:1151–64. doi: 10.4049/jimmunol.155.3.1151 7636184

[6] Putnam AL Vendrame F Dotta F Gottlieb PA. CD4+CD25high regulatory T cells in human autoimmune diabetes. J Autoimmun. (2005) 24:55–62. doi: 10.1016/j.jaut.2004.11.004 15725577

[7] Mhanna V Fourcade G Barennes P Quiniou V Pham HP Ritvo PG. Impaired activated/memory regulatory T cell clonal expansion instigates diabetes in NOD mice. Diabetes. (2021) 70:976–85. doi: 10.2337/db20-0896 33479057

[8] Tabarkiewicz J Piekarski R Wasiak M Wdowiak P Rolinski J Szewczyk L. Evaluation of circulating dendritic cells and T regulatory cells in children with newly diagnosed type 1 diabetes mellitus. Pediatric. Endocrinol. (2009) 8:27–34.

[9] Yue T Sun F Yang C Wang F Luo J Yang P. The AHR signaling attenuates autoimmune responses during the development of type 1 diabetes. Front Immunol. (2020) 11:1510. doi: 10.3389/fimmu.2020.01510 32849515 PMC7426364

[10] Gandhi R Kumar D Burns EJ Nadeau M Dake B Laroni A. Activation of the aryl hydrocarbon receptor induces human type 1 regulatory T cell-like and Foxp3(+) regulatory T cells. Nat Immunol. (2010) 11:846–53. doi: 10.1038/ni.1915 PMC2929008 20676092

[11] Zhu J Luo L Tian L Yin S Ma X Cheng S. Aryl hydrocarbon receptor promotes IL-10 expression in inflammatory macrophages through src-STAT3 signaling pathway. Front Immunol. (2018) 9:2033. doi: 10.3389/fimmu.2018.02033 30283437 PMC6156150

[12] Rodríguez-Sosa M Elizondo G López-Durán RM Rivera I Gonzalez FJ Vega L. Over production of IFN-gamma and IL-12 in AHR-null mice. FEBS Lett. (2005) 579:6403–10. doi: 10.1016/j.febslet.2005.10.023 16289099

[13] Stockinger B Shah K Wincent E. AHR in the intestinal microenvironment: safeguarding barrier function. Nat Rev Gastroenterol Hepatol. (2021) 18:559–70. doi: 10.1038/s41575-021-00430-8 PMC7611426 33742166

[14] Bell KJ Saad S Tillett BJ McGuire HM Bordbar S Yap YA. Metabolite-based dietary supplementation in human type 1 diabetes is associated with microbiota and immune modulation. Microbiome. (2022) 10:9. doi: 10.1186/s40168-021-01193-9 35045871 PMC8772108

[15] Kerkvliet NI Steppan LB Vorachek W Oda S Farrer D Wong CP. Activation of aryl hydrocarbon receptor by TCDD prevents diabetes in NOD mice and increases Foxp3+ T cells in pancreatic lymph nodes. Immunotherapy. (2009) 1:539–47. doi: 10.2217/imt.09.24 PMC2823486 20174617

[16] Yeste A Takenaka MC Mascanfroni ID Nadeau M Kenison JE Patel B. Tolerogenic nanoparticles inhibit T cell-mediated autoimmunity through SOCS2. Sci Signal. (2016) 9:ra61. doi: 10.1126/scisignal.aad0612 27330188

[17] Ehrlich AK Pennington JM Wang X Rohlman D Punj S Löhr CV. Activation of the aryl hydrocarbon receptor by 10-cl-BBQ prevents insulitis and effector T cell development independently of foxp3+ Regulatory T cells in nonobese diabetic mice. J Immunol. (2016) 196:264–73. doi: 10.4049/jimmunol.1501789 PMC4684970 26573835

[18] Kim YT Steinberg C. Immunologic studies on the induction of diabetes in experimental animals. Cellular basis for the induction of diabetes by streptozotocin. Diabetes. (1984) 33:771–7. doi: 10.2337/diab.33.8.771 6235141

[19] Amdare N Purcell AW DiLorenzo TP. Noncontiguous T cell epitopes in autoimmune diabetes: From mice to men and back again. J Biol Chem. (2021) 297:100827. doi: 10.1016/j.jbc.2021.100827 34044020 PMC8233151

[20] Jonić N Koprivica I Chatzigiannis CM Tsiailanis AD Kyrkou SG Tzakos EP. Development of fluoAHRL: A novel synthetic fluorescent compound that activates AHR and potentiates anti-inflammatory T regulatory cells. Molecules. (2024) 29:2988. doi: 10.3390/molecules29132988 38998940 PMC11243367

[21] Weigmann B Tubbe I Seidel D Nicolaev A Becker C Neurath MF. Isolation and subsequent analysis of murine lamina propria mononuclear cells from colonic tissue. Nat Protoc. (2007) 2:2307–11. doi: 10.1038/nprot.2007.315 17947970

[22] Schindelin J Arganda-Carreras I Frise E Kaynig V Longair M Pietzsch T. Fiji: an open-source platform for biological-image analysis. Nat Methods. (2012) 9:676–82. doi: 10.1038/nmeth.2019 PMC3855844 22743772

[23] Luo Z Soläng C Mejia-Cordova M Thorvaldson L Blixt M Sandler S. Kinetics of immune cell responses in the multiple low-dose streptozotocin mouse model of type 1 diabetes. FASEB Bioadv. (2019) 1:538–49. doi: 10.1096/fba.2019-00031 PMC6996374 32123849

[24] Marafini I Monteleone I Laudisi F Monteleone G. Aryl hydrocarbon receptor signalling in the control of gut inflammation. Int J Mol Sci. (2024) 25:4527. doi: 10.3390/ijms25084527 38674118 PMC11050475

[25] Qiu J Heller JJ Guo X Chen ZM Fish K Fu YX. The aryl hydrocarbon receptor regulates gut immunity through modulation of innate lymphoid cells. Immunity. (2012) 36:92–104. doi: 10.1016/j.immuni.2011.11.011 22177117 PMC3268875

[26] Nguyen NT Kimura A Nakahama T Chinen I Masuda K Nohara K. Aryl hydrocarbon receptor negatively regulates dendritic cell immunogenicity via a kynurenine-dependent mechanism. Proc Natl Acad Sci USA. (2010) 107:19961–6. doi: 10.1073/pnas.1014465107 PMC2993339 21041655

[27] Takenaka MC Robson S Quintana FJ. Regulation of the T cell response by CD39. Trends Immunol. (2016) 37:427–39. doi: 10.1016/j.it.2016.04.009 PMC5215082 27236363

[28] McDonald A Nicaise A Sears ER Bell A Kummari E Kaplan BLF. Potential for TCDD to induce regulatory functions in B cells as part of the mechanism for T cell suppression in EAE. Toxicol Appl Pharmacol. (2022) 454:116259. doi: 10.1016/j.taap.2022.116259 36179859 PMC10509645

[29] Zhang L Ma J Takeuchi M Usui Y Hattori T Okunuki Y. Suppression of experimental autoimmune uveoretinitis by inducing differentiation of regulatory T cells via activation of aryl hydrocarbon receptor. Invest. Ophthalmol Vis Sci. (2010) 51:2109–17. doi: 10.1167/iovs.09-3993 20007828

[30] Sorg O. AHR signalling and dioxin toxicity. Toxicol Lett. (2014) 230:225–33. doi: 10.1016/j.toxlet.2013.10.039 24239782

[31] Lin L Dai Y Xia Y. An overview of aryl hydrocarbon receptor ligands in the Last two decades, (2002-2022): A medicinal chemistry perspective. Eur J Med.Chem. (2022) 244:114845. doi: 10.1016/j.ejmech.2022.114845 36274276

[32] Quintana FJ Murugaiyan G Farez MF Mitsdoerffer M Tukpah AM Burns EJ. An endogenous aryl hydrocarbon receptor ligand acts on dendritic cells and T cells to suppress experimental autoimmune encephalomyelitis. Proc Natl Acad Sci USA. (2010) 107:20768–73. doi: 10.1073/pnas.1009201107 PMC2996442 21068375

[33] Schulze-Topphoff U Shetty A Varrin-Doyer M Molnarfi N Sagan SA Sobel RA. Laquinimod, a quinoline-3-carboxamide, induces type II myeloid cells that modulate central nervous system autoimmunity. PloS One. (2012) 7:e33797. doi: 10.1371/journal.pone.0033797 22479444 PMC3316495

[34] Jolivel V Luessi F Masri J Kraus SH Hubo M Poisa-Beiro L. Modulation of dendritic cell properties by laquinimod as a mechanism for modulating multiple sclerosis. Brain. (2013) 136:1048–66. doi: 10.1093/brain/awt023 23518712

[35] Ott M Avendaño-Guzmán E Ullrich. E Dreyer C Strauss J Harden M. Laquinimod, a prototypic quinoline-3-carboxamide and aryl hydrocarbon receptor agonist, utilizes a CD155-mediated natural killer/dendritic cell interaction to suppress CNS autoimmunity. J Neuroinflamm. (2019) 16:49. doi: 10.1186/s12974-019-1437-0 PMC6390632 30808363

[36] Li X Atkinson MA. The role for gut permeability in the pathogenesis of type 1 diabetes – a solid or leaky concept? Pediatr Diabetes. (2015) 16:485–92. doi: 10.1111/pedi.12305 PMC4638168 26269193

[37] Đedović N Paunović V Stojanović I. Isolation and enrichment of mouse insulin-specific CD4^+^ T regulatory cells. J Immunol Methods. (2019) 470:46–54. doi: 10.1016/j.jim.2019.04.011 31039339

[38] Sorini C Cosorich I Lo Conte M De Giorgi L Facciotti F Lucianò R. Loss of gut barrier integrity triggers activation of islet-reactive T cells and autoimmune diabetes. Proc Natl Acad Sci USA. (2019) 116:15140–9. doi: 10.1073/pnas.1814558116 PMC6660755 31182588

[39] Yuan X Tong B Dou Y Wu X Wei Z Dai Y. Tetrandrine ameliorates collagen-induced arthritis in mice by restoring the balance between Th17 and Treg cells via the aryl hydrocarbon receptor. Biochem Pharmacol. (2016) 101:87–99. doi: 10.1016/j.bcp.2015.11.025 26640276

[40] Guo Z Jang MH Otani K Bai Z Umemoto E Matsumoto M. CD4+CD25+ regulatory T cells in the small intestinal lamina propria show an effector/memory phenotype. Int Immunol. (2008) 20:307–15. doi: 10.1093/intimm/dxm143 18184698

[41] Kiss EA Vonarbourg C Kopfmann S Hobeika E Finke D Esser C. Natural aryl hydrocarbon receptor ligands control organogenesis of intestinal lymphoid follicles. Science. (2011) 334:1561–5. doi: 10.1126/science.1214914 22033518

[42] Lee JS Cella M McDonald KG Garlanda C Kennedy GD Nukaya M. AHR drives the development of gut ILC22 cells and postnatal lymphoid tissues via pathways dependent on and independent of Notch. Nat Immunol. (2011) 13:144–51. doi: 10.1038/ni.2187 PMC3468413 22101730

[43] Zhou L Chu C Teng F Bessman NJ Goc J Santosa EK. Innate lymphoid cells support regulatory T cells in the intestine through interleukin-2. Nature. (2019) 568:405–9. doi: 10.1038/s41586-019-1082-x PMC6481643 30944470

[44] Saksida T Paunović V Koprivica I Mićanović D Jevtić B Jonić N. Development of type 1 diabetes in mice is associated with a decrease in IL-2-producing ILC3 and foxP3+ Treg in the small intestine. Molecules. (2023) 28:3366. doi: 10.3390/molecules28083366 37110604 PMC10141349

[45] Mejía-León ME Barca AM. Diet, microbiota and immune system in type 1 diabetes development and evolution. Nutrients. (2015) 7:9171–84. doi: 10.3390/nu7115461 PMC4663589 26561831

[46] Pearson JA Kakabadse D Davies J Peng J Warden-Smith J Cuff S. Altered gut microbiota activate and expand insulin B15-23-reactive CD8+ T cells. Diabetes. (2019) 68:1002–13. doi: 10.2337/db18-0487 PMC6477900 30796028

[47] Stojanović I Saksida T Miljković Đ Pejnović N. Modulation of intestinal ILC3 for the treatment of type 1 diabetes. Front Immunol. (2021) 12:653560. doi: 10.3389/fimmu.2021.653560 34149694 PMC8209467

[48] Schneider A Long SA Cerosaletti K Ni CT Samuels P Kita M. In active relapsing-remitting multiple sclerosis, effector T cell resistance to adaptive T(regs) involves IL-6-mediated signaling. Sci Transl Med. (2013) 5:170ra15. doi: 10.1126/scitranslmed.3004970 23363979

[49] Xiao H Wang S Miao R Kan W. TRAIL is associated with impaired regulation of CD4+CD25- T cells by regulatory T cells in patients with rheumatoid arthritis. J Clin Immunol. (2011) 31:1112–9. doi: 10.1007/s10875-011-9559-x 21732015

[50] Lawson JM Tremble J Dayan C Beyan H Leslie RD Peakman M. Increased resistance to CD4^+^CD25^hi^ regulatory T cell-mediated suppression in patients with type 1 diabetes. Clin Exp Immunol. (2008) 154:353–9. doi: 10.1111/j.1365-2249.2008.03810.x PMC2633239 19037920

