# Supplementary material for: Novel AHR ligand AGT-5 ameliorates type 1 diabetes in mice through regulatory cell activation in the early phase of the disease
Source: Front Immunol. 2024 Sep 6;15:1454156. doi: 10.3389/fimmu.2024.1454156 (PMC11412818; doi:10.3389/fimmu.2024.1454156)
Supplement: Supplementary file 1 [file DataSheet1.docx]

Supplementary Material

**Novel AHR ligand AGT-5 ameliorates type 1 diabetes in mice through regulatory cell activation in the early phase of the disease**

*Natalija Jonić^1^, Ivan Koprivica^1^,* *Stavroula G. Kyrkou^2^, Vasileios-Panagiotis Bistas^2^, Christos Chatzigiannis^2^, Nataša Radulović^1^, Ivan Pilipović^1^, Andjelina Jovanović^3^, Milan B. Jovanović^3,4^, Mirjana Dimitrijević^1^, Andreas G. Tzakos^2,5^, Ivana Stojanović^1^*

**
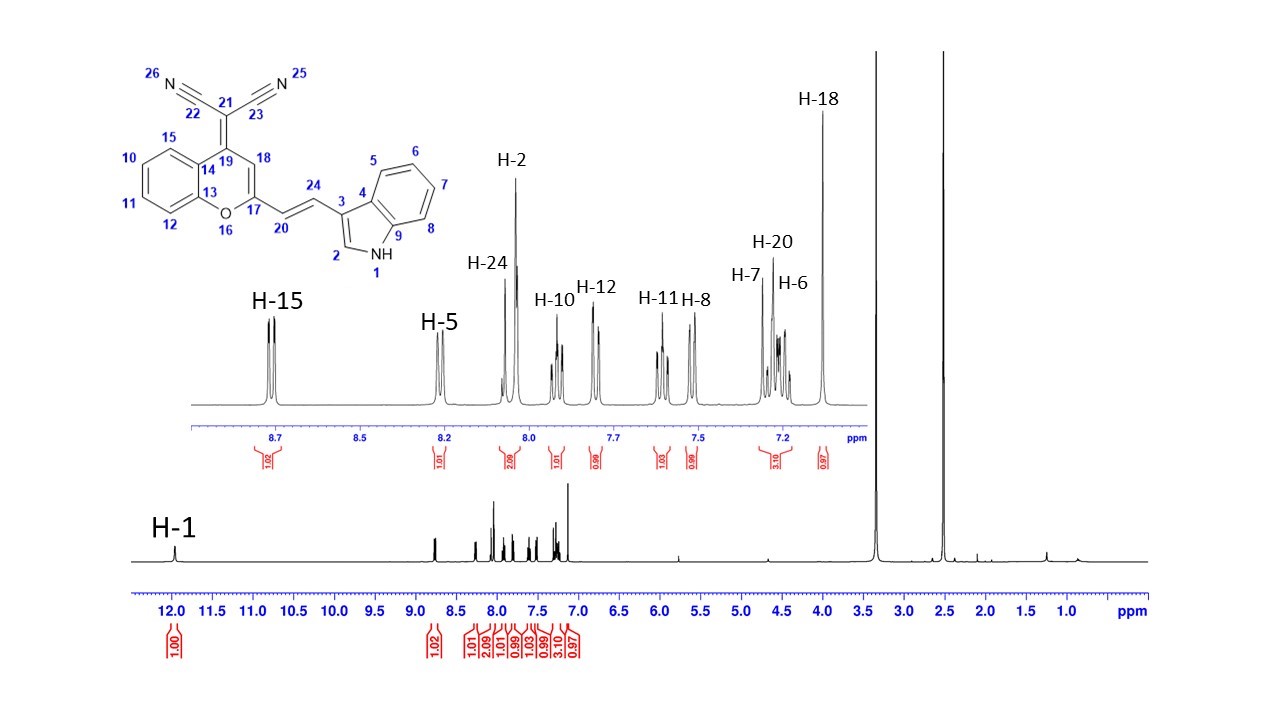
**

Figure S1. ^1^H-NMR spectra in DMSO-d_6_ of AGT-5 with magnification in the region of aromatic protons.

*
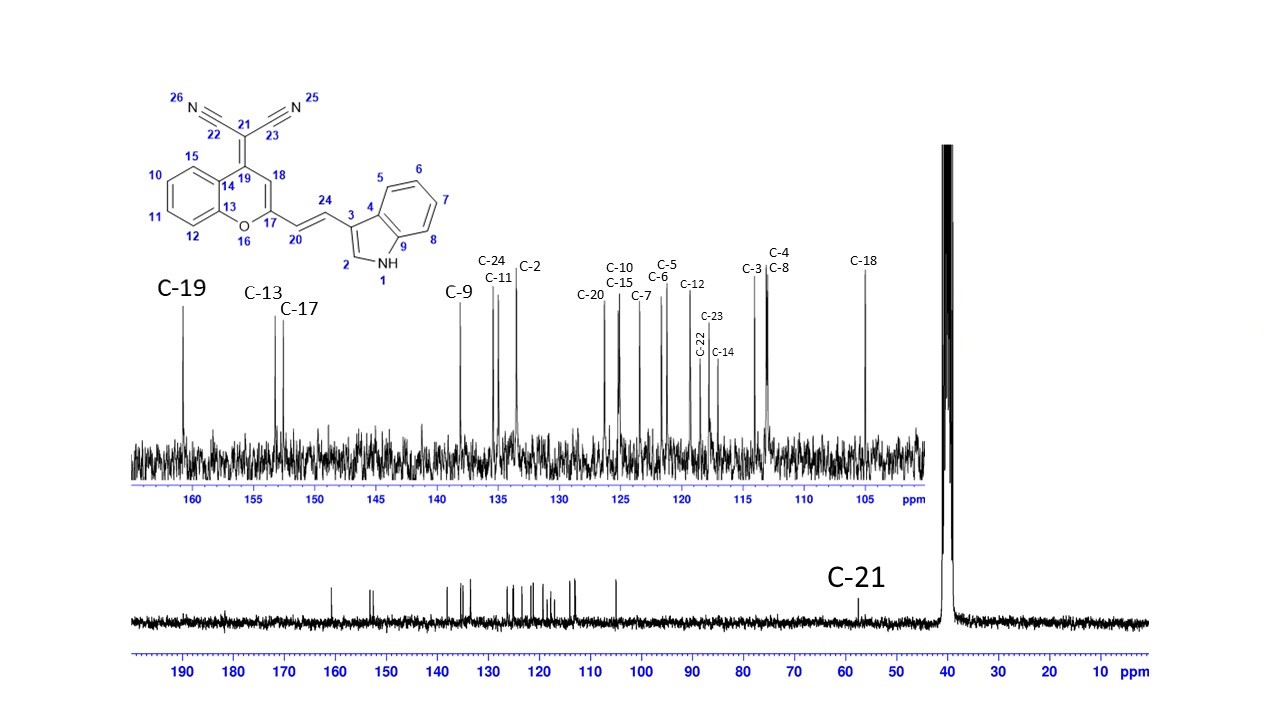
*

Figure S2. ^13^C-NMR spectra in DMSO-d_6_ of AGT-5.


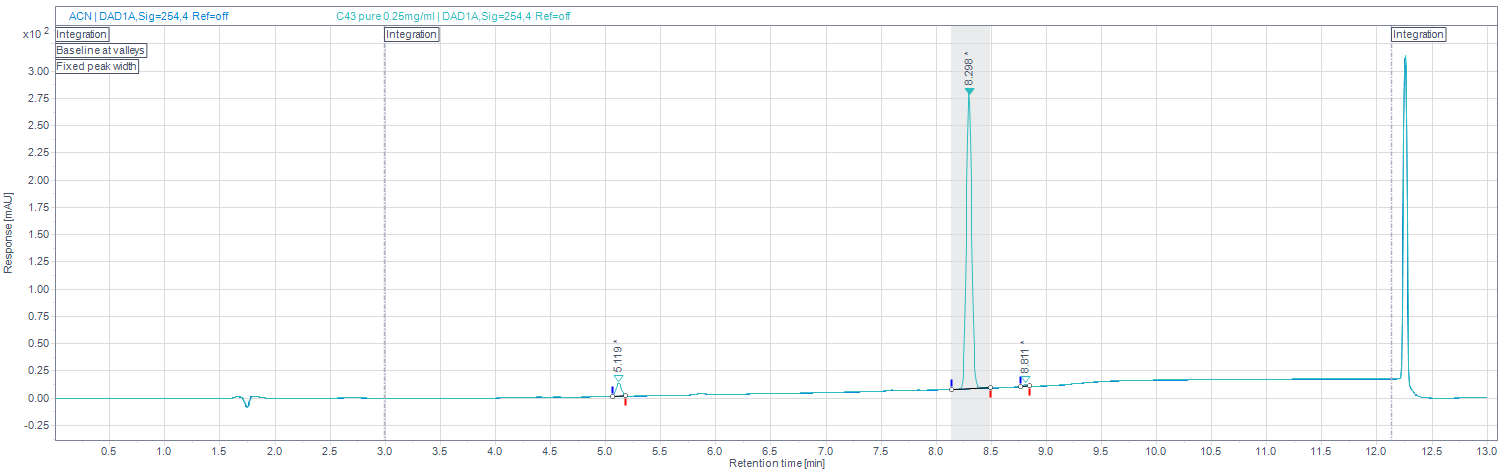


Figure S3. HPLC chromatogram of AGT-5 in acetonitrile (0.25 mg/ml), overlay with acetonitrile as blank, showing purity of 96%.


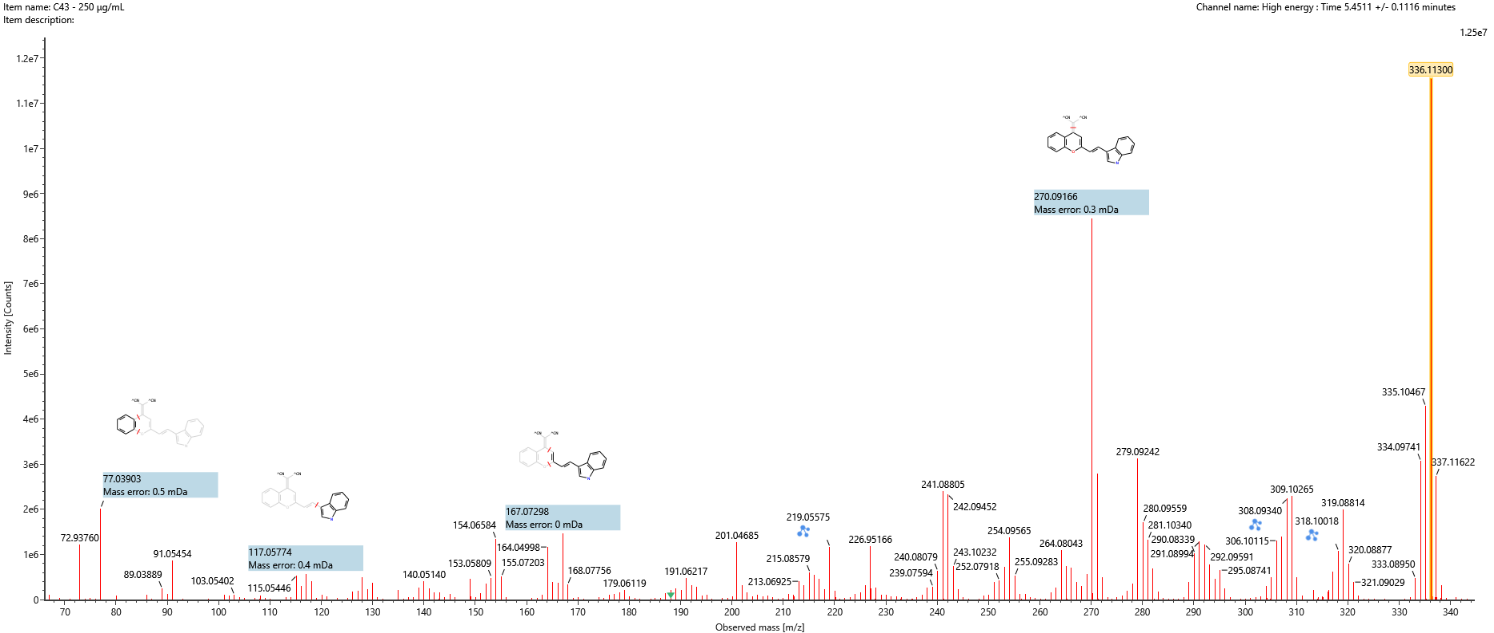


Figure S4. MS spectra of AGT-5 showing the desired m/z. [M+H] = 336,11300.


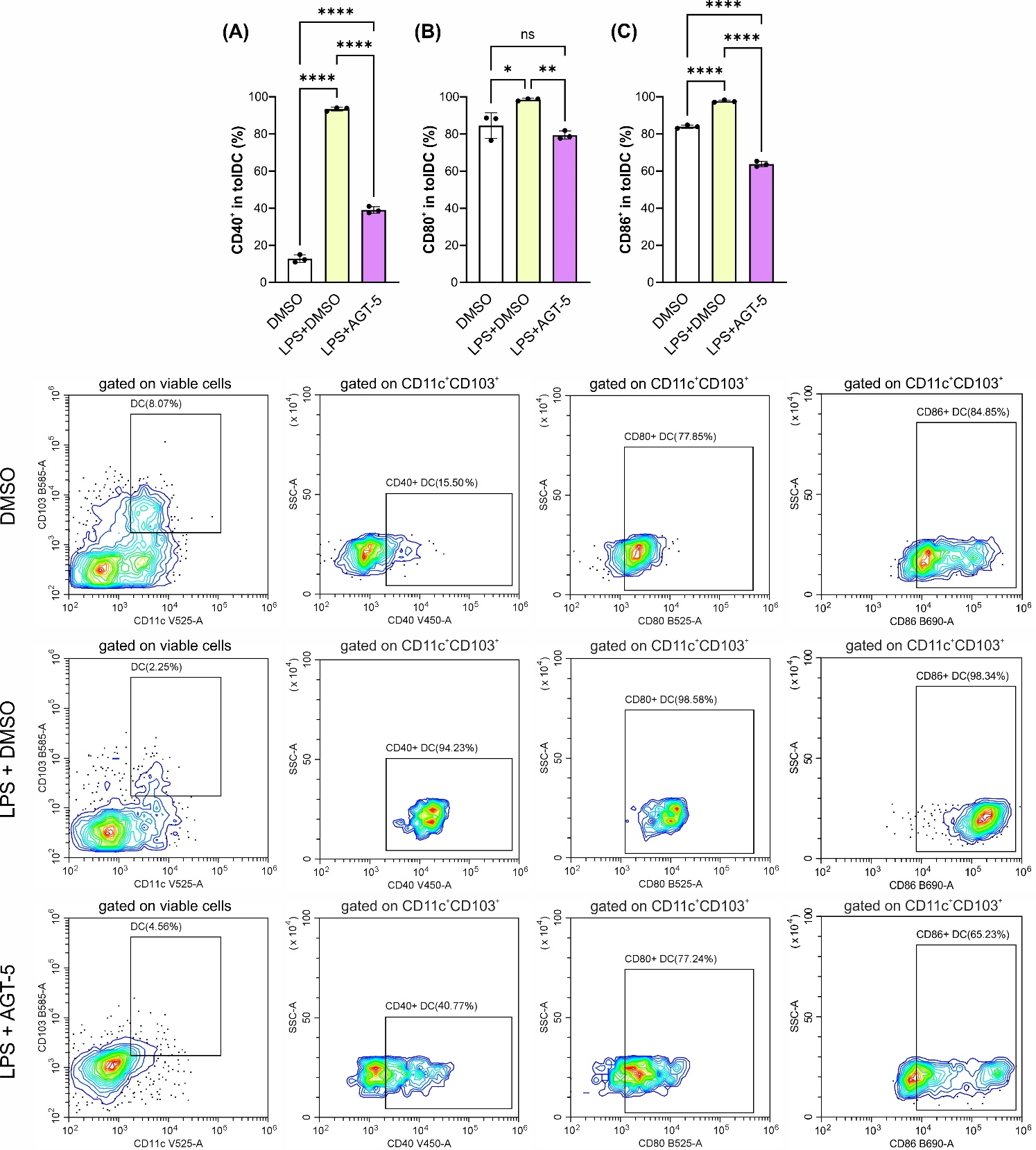


Figure S5. The effect of AGT-5 on DC *in vitro*. Bone-marrow derived DC were treated with DMSO, LPS+DMSO, or LPS+AGT-5 (0.75 µM) for 24 h. Histograms show the frequencies of cells expressing CD40 (A), CD80 (B) and CD86 (C) co-stimulatory molecules within tolerogenic DC (tolDC). Flow cytometry analysis of the proportions is shown. As indicated in the representative flow cytometry contour plots, cells were gated on viable population (according to FSC-A, SSC-A), then on CD11c^+^ and CD103^+^ cell population (tolDC), and then analyzed for the expression of co-stimulatory molecules. *p<0.05, **p<0.01, ****p<0.001. ns, not significant


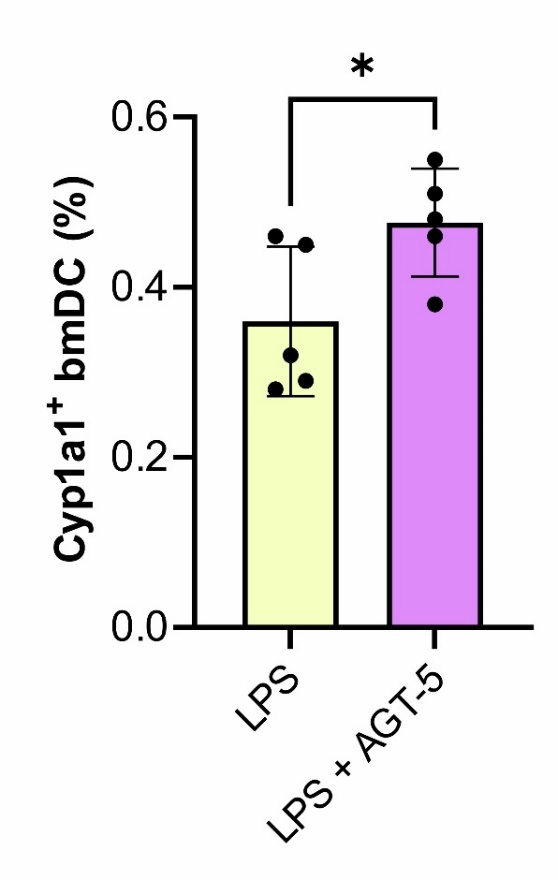


Figure S6. *In vitro* effect of AGT-5 on Cyp1a1 expression in DC. Bone-marrow derived DC (bmDC) were treated with LPS+DMSO or LPS+AGT-5 (0.75 µM) for 24 h. Histogram shows the frequencies of cells expressing Cyp1a1, determined by flow cytometry. *p<0.05 represents statistical significance in values between LPS and LPS+AGT-5-treated DC cultures.


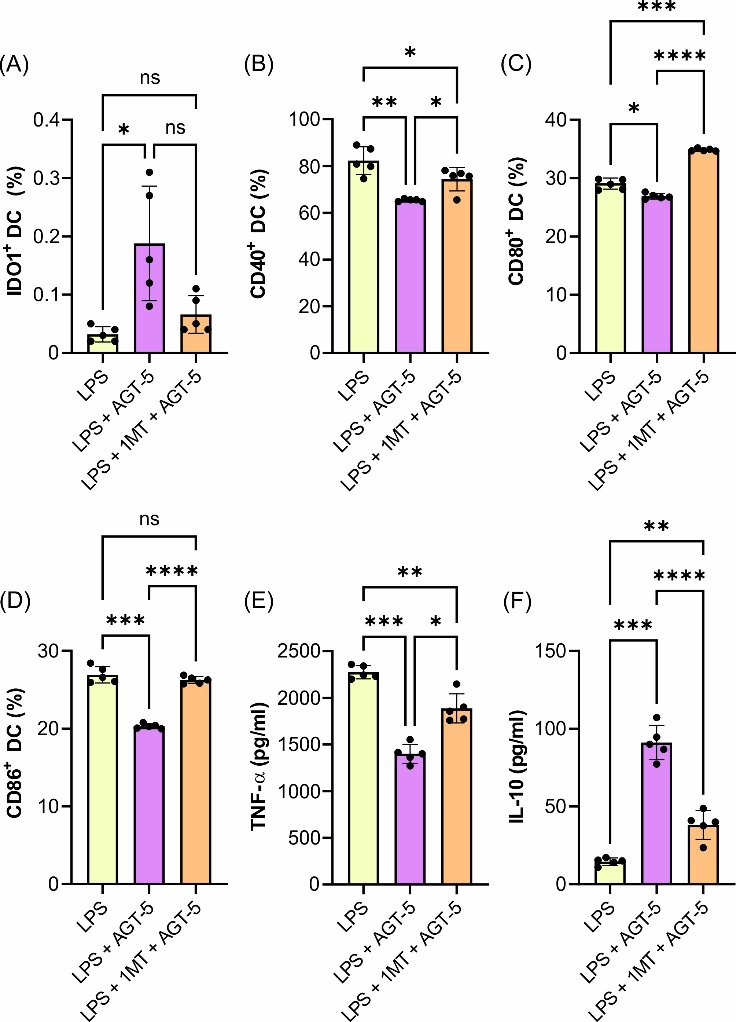


Figure S7. The effect of IDO1 inhibition on AGT-5 effects on DC *in vitro*. Bone-marrow derived DC were treated with LPS+DMSO, LPS+AGT-5 (0.75 µM) or LPS in the presence of 1-methyl tryptophan (1MT, 0.5 mM) and AGT-5 (0.75 µM) for 24 h. Histograms show the frequencies of cells expressing IDO1 (A), CD40 (B), CD80 (C) and CD86 (D), determined by flow cytometry. Supernatants from the indicated DC cultures were assayed for TNF (E) and IL-10 (F) by ELISA. *p<0.05, **p<0.01, ***p<0.005, ****p<0.001. ns, not significant.


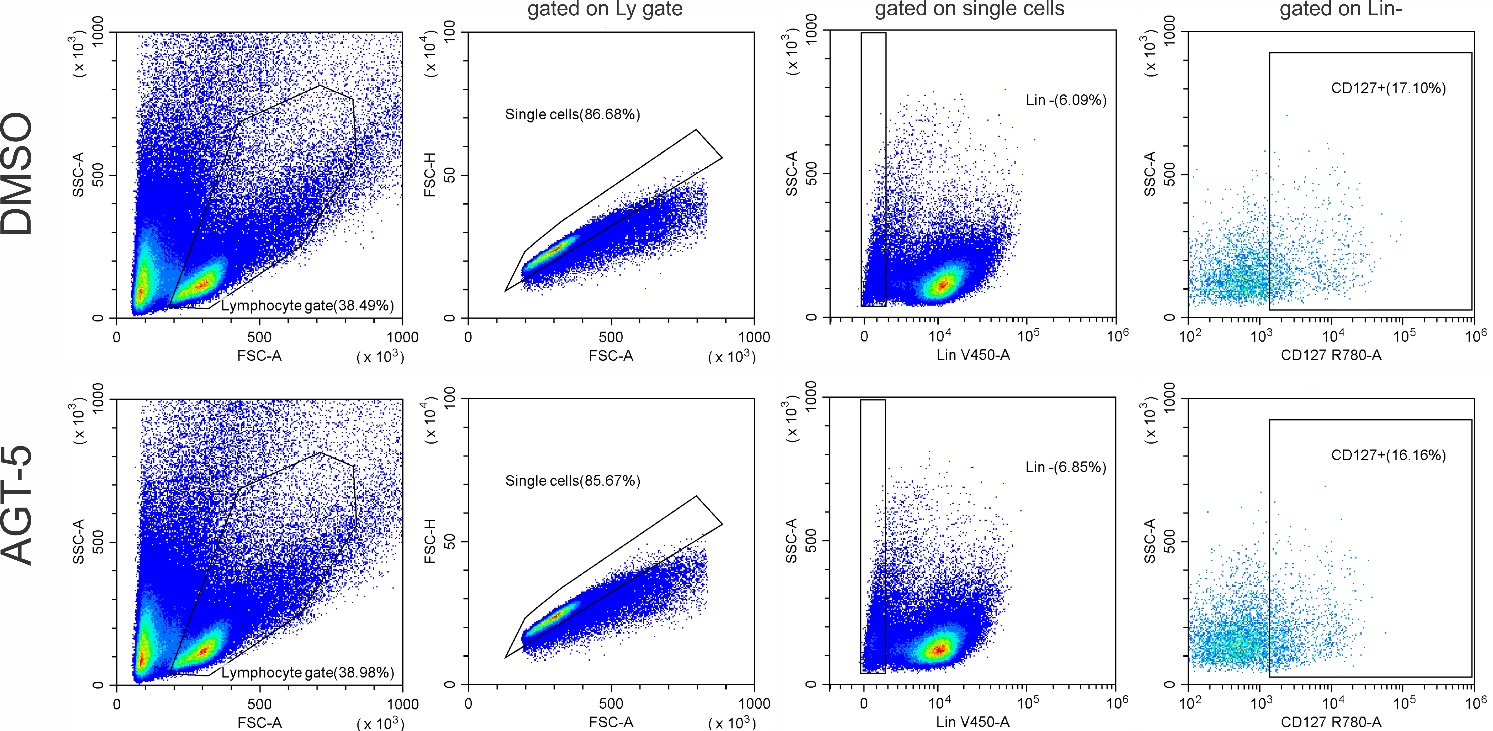


Figure S8. Gating strategy for human ILC3 in tonsil cell suspension after 48 h of incubation. Cells were first gated on lymphocyte gate (Ly), then on single cell gate, then on those negative for hematopoietic lineage markers (Lin^−^), and finally on CD127. Gating for CD294^−^ and CD117^+^ cells (ILC3) is shown in Figure 8.

| Table S1. Anti-mouse antibodies specific for surface molecules used in flow cytometry analysis | | | |
| --- | --- | --- | --- |
| **Antibody** | **Fluorophore** | **Host** | **Manufacturer** |
| CD45 | PE-Cy7 | Rat IgG2b, κ | Invitrogen, Waltham, MA, USA |
| Hematopoietic lineage antibody cocktail | FITC | Rat |  |
| CD127 | APC-eFluor™780 | Rat IgG2a, κ |  |
| CD4 | eFluor™506 | Rat IgG2a, κ |  |
| CD4 | PE-Cy7 | Rat IgG2b, κ |  |
| CD8 | APC-eFluor™780 | Rat IgG2a, κ |  |
| CD25 | PE | Rat IgG1, λ |  |
| CD25 | APC-eFluor™780 | Rat IgG1, λ |  |
| CYP1a1 | Polyclonal antibody | Rabbit IgG |  |
| CD11b | Alexa Fluor™488 | Rat IgG2b,κ |  |
| CD11b | APC-Cy7 | Rat IgG2b,κ |  |
| CD11c | eFluor™506 | Armenian hamster IgG |  |
| CD11c | PE-Cy5.5 | Armenian hamster IgG |  |
| CD80 | FITC | Armenian hamster IgG |  |
| CD86 | PE-Cy5 | Rat IgG2a,κ |  |
| CD40 | APC | Rat IgG2b, κ |  |
| MHC II | APC-eFluor™780 | Rat IgG2b,κ |  |
| CD39 | PE-eFluor™510 | Rat IgG2b,κ |  |
| CD73 | PE-Cy7 | Rat IgG1 |  |
| CD103 | PE | Armenian hamster IgG |  |
| CD103 | PE/Dazzle™594 | Armenian hamster IgG | BioLegend, San Diego, CA, USA |
| Anti-Rabbit IgG (H+L) Secondary Antibody | Alexa Fluor™488 | Goat | Invitrogen, Waltham, MA, USA |

| Table S2. Anti-mouse antibodies specific for intracellular molecules used in flow cytometry analysis | | | |
| --- | --- | --- | --- |
| **Antibody** | **Fluorophore** | **Host** | **Manufacturer** |
| Foxp3 | PE-Cy5.5 | Rat IgG2a, κ | Invitrogen, Waltham, MA, USA |
| RORγt | PE | Rat IgG2a, κ |  |
| Granzyme B | FITC | Rat IgG2a,κ |  |
| IDO1 | FITC | Rat IgG2b, κ |  |
| IFN-γ | APC | Rat IgG1, κ |  |
| IL-17 | PerCP-Cy5.5 | Rat IgG2a, κ |  |
| IL-17 | FITC | Rat IgG2a, κ |  |
| IL-2 | eFluor™450 | Rat IgG2b, κ |  |
| IL-22 | APC | Rat IgG2a, κ |  |
| IL-10 | PE | Rat IgG2b, κ |  |

| Table S3. Anti-human antibodies specific for surface and intracellular molecules staining used in Flow Cytometry | | | |
| --- | --- | --- | --- |
| **Antibody** | **Fluorophore** | **Host** | **Manufacturer** |
| CD80 | FITC | Mouse IgG1, κ | Invitrogen, Waltham, MA, USA |
| CD86 | PE-Cy5 | Mouse IgG2b, κ |  |
| Hematopoietic Lineage Antibody Cocktail | eFluor™450 | Mouse |  |
| CD117 ( c-Kit) | PE-Cy5 | Rat IgG2b, κ |  |
| CD127 | APC-eFluor™780 | Rat IgG2a, κ |  |
| CD294 (CRTH2) | APC | Rat IgG2a, κ |  |
| ILT3 | PE | Mouse IgG1, κ |  |
| HLA-DR | APC-Cy7 | Mouse IgG2a, κ | BioLegend, San Diego, CA, USA |
| CD83 | Biotin | Mouse IgG1, κ | MACS, Miltenyi Biotec, San Diego, CA, USA |
